# Prediction of lithium response using genomic data

**DOI:** 10.1038/s41598-020-80814-z

**Published:** 2021-01-13

**Authors:** William Stone, Abraham Nunes, Kazufumi Akiyama, Nirmala Akula, Raffaella Ardau, Jean-Michel Aubry, Lena Backlund, Michael Bauer, Frank Bellivier, Pablo Cervantes, Hsi-Chung Chen, Caterina Chillotti, Cristiana Cruceanu, Alexandre Dayer, Franziska Degenhardt, Maria Del Zompo, Andreas J. Forstner, Mark Frye, Janice M. Fullerton, Maria Grigoroiu-Serbanescu, Paul Grof, Ryota Hashimoto, Liping Hou, Esther Jiménez, Tadafumi Kato, John Kelsoe, Sarah Kittel-Schneider, Po-Hsiu Kuo, Ichiro Kusumi, Catharina Lavebratt, Mirko Manchia, Lina Martinsson, Manuel Mattheisen, Francis J. McMahon, Vincent Millischer, Philip B. Mitchell, Markus M. Nöthen, Claire O’Donovan, Norio Ozaki, Claudia Pisanu, Andreas Reif, Marcella Rietschel, Guy Rouleau, Janusz Rybakowski, Martin Schalling, Peter R. Schofield, Thomas G. Schulze, Giovanni Severino, Alessio Squassina, Julia Veeh, Eduard Vieta, Thomas Trappenberg, Martin Alda

**Affiliations:** 1 Faculty of Computer Science, Dalhousie University, Halifax, NS Canada; 2 Department of Psychiatry, Dalhousie University, Halifax, NS Canada; 3 Department of Biological Psychiatry and Neuroscience, Dokkyo Medical University School of Medicine, Mibu, Tochigi Japan; 4 National Institute of Mental Health, Bethesda, USA; 5 Unit of Clinical Pharmacology, University Hospital of Cagliari, Cagliari, Italy; 6 Department of Psychiatry, University of Geneva, Geneva, Switzerland; 7 Department of Psychiatry, University of Geneva Hospitals, Geneva, Switzerland; 8 Department of Clinical Neuroscience, the Centre for Psychiatric Research, Karolinska Institutet, Stockholm, Sweden; 9 Department of Molecular Medicine and Surgery, Karolinska Institutet, Stockholm, Sweden; 10 Karolinska University Hospital, Center for Molecular Medicine, Stockholm, Sweden; 11 Department of Psychiatry and Psychotherapy, Medical Faculty, Technische Universität Berlin, Dresden, Germany; 12 Université Paris Diderot, Paris, France; 13 Inserm, U1144, Team 1, Paris, France; 14 Department of Psychiatry, McGill University, Montreal, Canada; 15 Department of Psychiatry, National Taiwan University Hospital, Taipei, Taiwan; 16 Department of Translational Research, Max Planck Institute of Psychiatry, Munich, Germany; 17 Department of Basic Neurosciences, University of Geneva, Geneva, Switzerland; 18 Institute of Human Genetics, School of Medicine, University Hospital Bonn, University of Bonn, Bonn, Germany; 19 Department of Biomedical Sciences, University of Cagliari, Cagliari, Italy; 20 Centre for Human Genetics, University of Marburg, Marburg, Germany; 21 Department of Psychiatry, Mayo Clinic, Rochester, USA; 22 School of Psychiatry, University of New South Wales, Sydney, Australia; 23 Biometric Psychiatric Genetics Research Unit, Alexandru Obregia Clinical Psychiatric Hospital, Bucharest, Romania; 24 Mood Disorders Center Ottawa, Ottawa, Canada; 25 Department of Pathology of Mental Diseases, National Institute of Mental Health, Tokyo, Japan; 26 Department of Psychiatry, Osaka University, Osaka, Japan; 27 Hospital Clinic, University of Barcelona, Barcelona, Spain; 28 Institut d’Investigacio Biomedica August Pi i Sunyer, Barcelona, Spain; 29 Centro de Investigación Biomédica en Red de Salud Mental (CIBERSAM), Barcelona, Spain; 30 Laboratory for Molecular Dynamics of Mental Disorders, RIKEN Center for Brain Science, Wako, Japan; 31 Department of Psychiatry, UCSD, San Diego, CA USA; 32 Department of Psychiatry, Psychotherapy and Psychosomatic Medicine, University Hospital of Frankfurt, Frankfurt am Main, Germany; 33 Department of Psychiatry, Psychosomatic Medicine and Psychotherapy, University Hospital of Würzburg, Würzburg, Germany; 34 Institute of Epidemiology and Preventive Medicine, National Taiwan University, Taipei, Taiwan; 35 Department of Public Health, National Taiwan University, Taipei, Taiwan; 36 Department of Psychiatry, Hokkaido University Graduate School of Medicine, Sapporo, Japan; 37 Department of Medical Sciences and Public Health, University of Cagliari, Cagliari, Italy; 38 Department of Pharmacology, Dalhousie University, Halifax, NS Canada; 39 Department of Psychiatry, University of Wurzburg, Würzburg, Germany; 40 Institute of Human Genetics, University of Bonn, Bonn, Germany; 41 Department of Psychiatry, Nagoya University Graduate School of Medicine, Nagoya, Japan; 42 Department of Genetic Epidemiology in Psychiatry, Central Institute of Mental Health, Medical Faculty Mannheim, Heidelberg University, Mannheim, Germany; 43 Montreal Neurological Institute, McGill University, Montreal, Canada; 44 Department of Adult Psychiatry, Poznan University of Medical Sciences, Poznan, Poland; 45 Institute of Psychiatric Phenomics and Genomics, University of Munich, Munich, Germany; 46 Department of Child and Adolescent Psychiatry, Psychosomatics and Psychotherapy, University Hospital Essen, Essen, Germany

**Keywords:** Genetics, Medical research

## Abstract

Predicting lithium response prior to treatment could both expedite therapy and avoid exposure to side effects. Since lithium responsiveness may be heritable, its predictability based on genomic data is of interest. We thus evaluate the degree to which lithium response can be predicted with a machine learning (ML) approach using genomic data. Using the largest existing genomic dataset in the lithium response literature (n = 2210 across 14 international sites; 29% responders), we evaluated the degree to which lithium response could be predicted based on 47,465 genotyped single nucleotide polymorphisms using a supervised ML approach. Under appropriate cross-validation procedures, lithium response could be predicted to above-chance levels in two constituent sites (Halifax, Cohen’s kappa 0.15, 95% confidence interval, CI [0.07, 0.24]; and Würzburg, kappa 0.2 [0.1, 0.3]). Variants with shared importance in these models showed over-representation of postsynaptic membrane related genes. Lithium response was not predictable in the pooled dataset (kappa 0.02 [− 0.01, 0.04]), although non-trivial performance was achieved within a restricted dataset including only those patients followed prospectively (kappa 0.09 [0.04, 0.14]). Genomic classification of lithium response remains a promising but difficult task. Classification performance could potentially be improved by further harmonization of data collection procedures.

## Introduction

Bipolar disorder (BD) is a neuropsychiatric illness characterized by recurrent episodes of mania and depression. It is associated with a significant risk of suicide^1^—highest in early course of the illness—with many patients receiving effective treatment only after as many as 10 years^2^. One contributing factor to this lag is the number of medication trials that are typically undertaken to find the optimal treatment for a given patient. Since medication trials are based on an empirical approach (i.e. by trial-and-error), it may be possible to reduce this delay by predicting the best treatment for a given individual *a priori*. However, we currently lack predictive markers for this purpose.

Genomic data may be useful for the prediction of lithium responsiveness, given the familial aggregation of BD (particularly the lithium responsive type) among lithium responders^3^. At present, the best evidence of genomic correlates of lithium response is from the largest genome-wide association study (GWAS) of lithium response to date^4^. This study yielded a single associated locus on chromosome 21. Although the authors demonstrated that the response-related alleles were associated with lower rates of relapse in an independent sample of 73 patients treated for 2 years of lithium monotherapy, the out of sample predictive power of the genomic data overall remains unknown. This is due to the fact that GWAS is (A) not designed to evaluate predictive capacity, and (B) cannot account for epistatic effects.

Thus, the primary objective of the present study was to evaluate the degree to which lithium response can be predicted with a machine learning (ML) approach—which explicitly tackles the question of out-of-sample predictive power—using only genetic data. To do this, we employ the largest-ever set of genomic data from BD patients treated with lithium for maintenance therapy over a duration of at least one year, from 14 international site members of the Consortium on Lithium Genetics (ConLiGen). We also had two secondary objectives. First, we sought to evaluate–through pathway analysis–whether any above-chance classification performance was informed by genetic variants in specific biological pathways. Second, we sought to explore factors that might limit classification performance in this large multi-site dataset, such as assessment method (prospective follow-up vs. cross-sectional) and strength of lithium response.

## Methods

### Data collection

For this work we use the largest-ever set of genomic data from BD patients treated with lithium for maintenance therapy over a duration of at least one year, from 14 international site members of the Consortium on Lithium Genetics (ConLiGen). A detailed description of the data collection procedure was given by Hou et al.^4^. Briefly, a sample of 3013 patients were evaluated for long term response to lithium in one of 22 collaborating centres. For these analyses, we limited the feature set to the genotyped (non-imputed) SNPs that overlapped between all platforms. The same quality control measures described in the supplementary materials of Hou et al.^4^ were employed. We also removed data from sites that had either fewer than ten responders or fewer than 50 subjects in total. Table 1 provides a list of contributing sites and their relative proportions of lithium responders and non-responders. After quality control, our data included 2,210 subjects and 47,465 SNPs all from a total of 14 different centres. Further details of the methods are provided in the supplementary materials.

**Table 1:** Distributions of cases (lithium responders) and controls (non-responders) across sites.

Institution	Responders	Non-responders
University of Cagliari, Italy	55 (28%)	141 (72%)
Dalhousie University, Canada	159 (45%)	194 (55%)
University of NSW, Australia	13 (20%)	50 (80%)
Poznan University of Medical Sciences, Poland	47 (48%)	50 (52%)
UC San Diego, USA	23 (11%)	192 (89%)
RIKEN Brain Institute, Japan	31 (24%)	97 (76%)
Mayo Clinic, USA	22 (23%)	72 (77%)
University of Würzburg, Germany	30 (17%)	145 (83%)
Karolinska Institutet, Sweden	138 (45%)	166 (55%)
National Taiwan University, Taiwan	13 (14%)	79 (86%)
Obregia Hospital, Romania	32 (21%)	120 (79%)
University of Geneva, Switzerland	13 (23%)	44 (77%)
University of Barcelona, Spain	20 (27%)	54 (73%)
INSERM, France	38 (18%)	172 (82%)
ALL	634 (29%)	1576 (71%)

### Classification analyses

Analyses were performed in four stages: (A) analysis of pooled data (henceforth the aggregate analysis), (B) analysis of individual site-level data (henceforth the site-level analysis), (C) an analysis in which we trained classifiers using data from all but one site, which was used as a validation set (henceforth the predict-one-site-out analysis), and (D) an analysis in which we repeated the aggregate analysis after leaving each site out, one at a time (henceforth the leave-one-site-out analysis).

As in the study by Hou et al.^4^, we define a lithium responder as a subject with Alda score $\ge 7$ in our primary classification analyses. However, it is possible that subjects whose scores are on the scale’s extremes are better exemplars of lithium response and non-response, respectively. Subjects who are more representative of the underlying phenotype might be more easily separable. To test this hypothesis, we conducted a supplementary experiment in which we repeated the classification analyses in the following four conditions: removing subjects with scores of (A) 6, then (B) {6,7}, then (C) {5,6,7}, and finally (D) {4,5,6,7,8}.

It is possible that the mode of follow-up (prospective vs. not) could influence the labeling of patients as lithium responders or non-responders. Thus, we split the data into two sets: the first included data from sites where patients were followed prospectively (Poznan, Cagliari, Halifax, Romania, Barcelona), and the second included only patients assessed retrospectively or cross-sectionally (Japan, Würzburg, Geneva, Paris, San Diego, Mayo, New South Wales, Karolinska, Taiwan). Within each of these datasets, we repeated the aggregate, leave-one-site-out, and predict-one-site-out analyses under the same cross-validation scheme as our other analyses.

### Classifiers

To provide an indication of the degree to which classification performance was sensitive to classifier architecture, we implemented two classification models: L2-penalized logistic regression (LR) and extreme gradient boosted trees.

Logistic regression is a simple method that learns a linear decision boundary between classes. Given the high dimensionality of the feature space and the fact that we expect there to be relatively few features that carry predictive capacity, we add an L2 regularization penalty to the log-likelihood loss function according to the default parameterization in the SciKit-Learn v.0.20.1 package^5^.

The XGBoost (extreme gradient boosting, XGB) algorithm^6^, similar to a random forest classifier^7^, pools a number of weak classifiers (decision trees) in order to create a single strong classifier with a reduced variance in comparison to the weak classifiers. On top of this, the XGB algorithm trains subsequent trees including information regarding mistakes that previous trees made (specifically, the residuals between predicted and ground truth values) which serves to reduce bias in the strong classifier. We use the python implementation of XGB from the distributed machine learning community (DMLC, https://github.com/dmlc/xgboost) and use the default parameters in all of our experiments.

### Model criticism

All analyses employed five-fold cross-validation stratified by lithium response. We did this to ensure that model performance is measured out of sample, thus minimizing the possibility of over fitting. By definition, the predict-one-site-out analysis validation phase is conducted out of sample.

Performance measures included accuracy, area under the receiver operator characteristic curve (AUC), sensitivity, specificity, positive predictive value (PPV), negative predictive value (NPV), F-1 score (F1), and Cohen’s kappa. Our primary outcome measure of interest was Cohen’s kappa, which is generally more conservative under class imbalance.

For the Cohen’s kappa metric, we simulated *p* values that represented the probability *p* that a “trivial” or “null” classifier applied to a data set with the same proportion of positive examples would achieve greater performance. For the experiments in which we perform a stratified k-fold cross-validation, we combine the results from each testing set before applying this technique. Mathematical details for this procedure are provided in the Appendix.

### Feature importance and gene set analysis

For the best classified sites within the site-level analysis, we first compared the feature importance values (or LR coefficients) between classifiers trained on a given site. This was done to evaluate whether above-chance classification performance obtained with different models was attributable to the same features. For models in which multiple sites were classified with above-chance performance, we compared the feature importance/coefficient values between those sites. In both cases, we computed the mean feature importances/coefficients (model specific) over the respective site’s cross-validation folds. The expected values were then compared using Kendall’s Tau (using the Scipy v. 1.3.0 implementation). Where we compared feature importances from the XGBoost algorithm (which are not directional) against LR coefficients, we did so using the absolute value of the LR coefficients. However, when comparing coefficients between LR models (i.e. one trained on site A, and the other on site B), no such transformation was made. We report the tau statistic and *p* value, with the statistical significance threshold set to $\alpha =0.0125$ (Bonferroni correction for 4 comparisons).

To determine a within site feature importance ranking for the LR classifier for gene ontology analysis^8^ on the Dalhousie sample, we first computed the median LR coefficient (across the cross-validation folds) for all SNPs whose LR coefficients had the same sign across folds. We then ranked SNPs by the absolute value of the median of their regression coefficients and selected the top quartile (hereafter referred to as the effect set) for gene ontology analyses. Next, we used the biopython package^9^ to determine the gene(s) with which each SNP in the data set is associated (if any) and separated the effect set of genes from the total (reference) set. We then compare the reference and effect sets using the statistical overrepresentation test in PANTHER^8^. To limit the penalty incurred by multiple comparisons, in both the effect and reference gene lists we omitted all duplicates and genes that were either uncharacterized or not catalogued by PANTHER.

We investigated the overlap in feature importance for LR classifiers trained on the Dalhousie and Würzburg samples separately. In this scenario, we found the intersection of all SNPs whose LR coefficients matched in sign between both sites and took this to be the effect set. We then subjected the genes associated with these SNPs to the same gene set analysis outlined above.

## Results

### Demographic statistics

Demographic statistics are reported in Table 2. There were no statistically significant differences in sex, diagnosis, or age at onset between lithium responders and non-responders. There was a small but statistically significant difference in the presence of psychosis (721/1576 = 0.45 among non-responders, and 253/634 = 0.40 among responders; *p* = 0.001).

**Table 2:** Demographic statistics.

Variable	Non-responders	Responders	*p* value
n	1576	634	
Female (%)	899 (57)	377 (59.5)	0.32
DSM diagnosis (%)			0.56
Bipolar I	1230 (78)	490 (77.3)	
Bipolar II	338 (21.4)	143 (22.6)	
Schizoaffective	4 (0.3)	0 (0)	
Bipolar NOS	4 (0.3)	1 (0.2)	
Age of onset (years)	28.7 (11.3)	25.5 (10.7)	0.64
History of psychosis (%)	721 (45.7)	253 (40)	0.001

Categorical variables are presented as counts and percentages, whereas continuous variables are presented as means and standard deviations (SD). History of psychosis refers to the occurrence of psychotic symptoms at any point during the course of illness. Abbreviations: age at onset (AAO), not otherwise specified (NOS).

### Aggregate analysis

**Figure 1:**
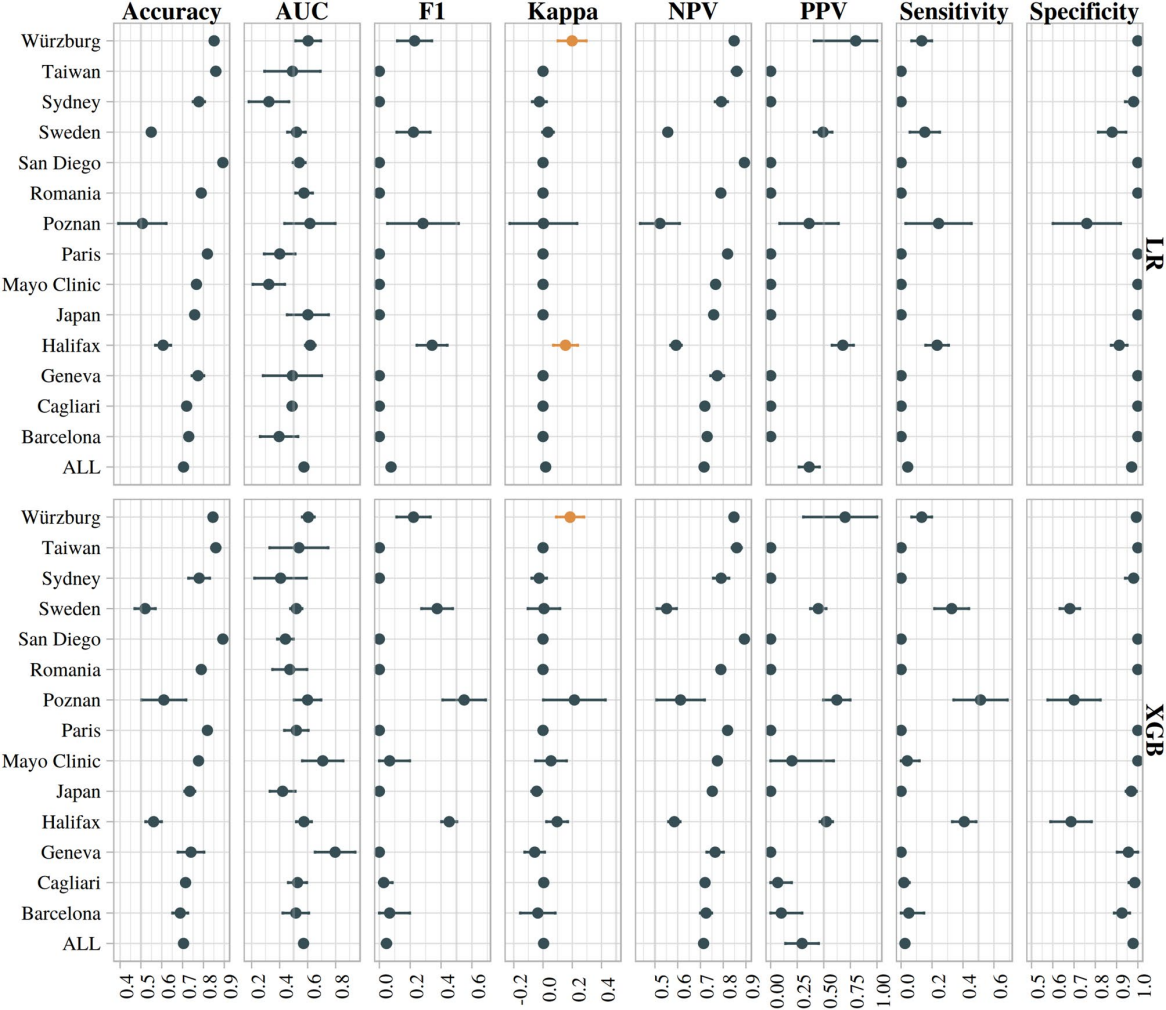
Performance of the logistic regression (LR), and XGBoost (XGB) classifiers on the aggregate and site-level analyses. Faceting of plots along columns corresponds to different classification statistics. Faceting along rows corresponds to the aforementioned classification algorithms. Within each plot, the x-axis represents the statistic value, and the y-axis corresponds to the dataset under which the classification analysis was performed. Plot markers denote the mean performance over the respective number of cross-validation folds, with error bars denoting the empirical 95% confidence intervals. In the column for Kappa, where the *p* value from the criticism analysis fell below 0.01 in comparison to the null classifier we coloured the point yellow to represent exceedance of chance; all other points are coloured dark blue. Abbreviations: all sites (ALL; i.e. aggregate analysis), area under the receiver operating characteristic curve (AUC), positive predictive value (PPV), negative predictive value (NPV), F-1 score (F1).

Figure 1 and Tables 3 and S1 (in Appendix; for the XGBoost classifier) report classification performance for each classifier for the aggregate analysis. On the pooled data, neither the LR (kappa 0.02 [− 0.01, 0.04], *p* = 0.2) nor XGBoost (kappa 0 [− 0.02, 0.03], *p* = 0.4) classifiers obtained classification performance in exceedance of chance.

**Table 3:** Performance of the logistic regression (LR) classifier on the aggregate and site-level analyses.

Centre (LR+/LR−; %)	AUC	Accuracy	F1	NPV	PPV	Sensitivity	Specificity	Kappa
ALL (29/71)	0.57 (0.55, 0.6)	0.7 (0.7, 0.71)	0.08 (0.05, 0.1)	0.72 (0.71, 0.72)	0.36 (0.27, 0.46)	0.04 (0.03, 0.06)	0.97 (0.97, 0.98)	0.02 (− 0.01, 0.04)
Halifax (45/55)	0.62 (0.58, 0.65)	0.61 (0.57, 0.64)	0.34 (0.24, 0.44)	0.59 (0.57, 0.62)	0.68 (0.58, 0.78)	0.23 (0.16, 0.31)	0.91 (0.87, 0.95)	0.15 (0.07, 0.24)^a^
Würzburg (17/83)	0.6 (0.51, 0.69)	0.85 (0.84, 0.86)	0.23 (0.12, 0.34)	0.85 (0.84, 0.86)	0.8 (0.41, 1.0)	0.13 (0.07, 0.2)	1.0 (1.0, 1.0)	0.2 (0.1, 0.3)^a^
Barcelona (27/73)	0.4 (0.26, 0.53)	0.73 (0.72, 0.74)	0.0 (0.0, 0.0)	0.73 (0.72, 0.74)	0.0 (0.0, 0.0)	0.0 (0.0, 0.0)	1.0 (1.0, 1.0)	0.0 (0.0, 0.0)
Cagliari (28/72)	0.49 (0.46, 0.52)	0.72 (0.72, 0.72)	0.0 (0.0, 0.0)	0.72 (0.72, 0.72)	0.0 (0.0, 0.0)	0.0 (0.0, 0.0)	1.0 (1.0, 1.0)	0.0 (0.0, 0.0)
Geneva (23/77)	0.49 (0.28, 0.7)	0.77 (0.74, 0.8)	0.0 (0.0, 0.0)	0.77 (0.74, 0.8)	0.0 (0.0, 0.0)	0.0 (0.0, 0.0)	1.0 (1.0, 1.0)	0.0 (0.0, 0.0)
Japan (24/76)	0.6 (0.45, 0.75)	0.76 (0.75, 0.77)	0.0 (0.0, 0.0)	0.76 (0.75, 0.77)	0.0 (0.0, 0.0)	0.0 (0.0, 0.0)	1.0 (1.0, 1.0)	0.0 (0.0, 0.0)
Mayo (23/77)	0.32 (0.21, 0.44)	0.77 (0.75, 0.78)	0.0 (0.0, 0.0)	0.77 (0.75, 0.78)	0.0 (0.0, 0.0)	0.0 (0.0, 0.0)	1.0 (1.0, 1.0)	0.0 (0.0, 0.0)
Paris (18/82)	0.4 (0.29, 0.51)	0.82 (0.81, 0.83)	0.0 (0.0, 0.0)	0.82 (0.81, 0.83)	0.0 (0.0, 0.0)	0.0 (0.0, 0.0)	1.0 (1.0, 1.0)	0.0 (0.0, 0.0)
Poznan (48/52)	0.62 (0.44, 0.8)	0.51 (0.39, 0.62)	0.28 (0.05, 0.51)	0.52 (0.44, 0.61)	0.36 (0.08, 0.64)	0.24 (0.03, 0.45)	0.76 (0.6, 0.92)	0.0 (− 0.23, 0.23)
Romania (21/79)	0.57 (0.51, 0.64)	0.79 (0.78, 0.8)	0.0 (0.0, 0.0)	0.79 (0.78, 0.8)	0.0 (0.0, 0.0)	0.0 (0.0, 0.0)	1.0 (1.0, 1.0)	0.0 (0.0, 0.0)
San Diego (11/89)	0.54 (0.5, 0.58)	0.89 (0.88, 0.9)	0.0 (0.0, 0.0)	0.89 (0.88, 0.9)	0.0 (0.0, 0.0)	0.0 (0.0, 0.0)	1.0 (1.0, 1.0)	0.0 (0.0, 0.0)
Sweden (25/55)	0.52 (0.46, 0.58)	0.55 (0.54, 0.56)	0.22 (0.11, 0.33)	0.56 (0.54, 0.57)	0.49 (0.41, 0.58)	0.15 (0.06, 0.25)	0.88 (0.81, 0.94)	0.03 (− 0.0, 0.07)
Sydney (20/80)	0.32 (0.18, 0.47)	0.78 (0.75, 0.81)	0.0 (0.0, 0.0)	0.79 (0.76, 0.82)	0.0 (0.0, 0.0)	0.0 (0.0, 0.0)	0.98 (0.94, 1.0)	− 0.03 (− 0.07, 0.02)
Taiwan (14/86)	0.49 (0.29, 0.69)	0.86 (0.84, 0.88)	0.0 (0.0, 0.0)	0.86 (0.84, 0.88)	0.0 (0.0, 0.0)	0.0 (0.0, 0.0)	1.0 (1.0, 1.0)	0.0 (0.0, 0.0)

Table columns represent different classification statistics and values represent the mean of each statistic over five folds along with an empirical 95% confidence interval. In the column listing respective centres, we have included the percentage of lithium responders (LR+) and non-responders (LR−) in the format %LR+/%LR− to provide context for the classification results. Abbreviations: all sites (ALL; i.e. aggregate analysis), area under the receiver operating characteristic curve (AUC), positive predictive value (PPV), negative predictive value (NPV), F-1 score (F1).

^a^Kappa value was found to have a *p* value less than 0.01 in comparison to the null classifier.

### Site-level analyses

Figure 1 and Tables 3 and S1 (in Appendix; for the XGBoost classifier) also report the classification performance of each classifier in the site-level analysis. The LR classifier with the Würzburg sample (17% responders and 83% non-responders) achieved an accuracy of 0.85 (95% CI [0.84, 0.86]), AUC-ROC 0.6 (0.51–0.69), sensitivity 0.13 (0.07–0.2), specificity 1 (1–1), PPV of 0.8 (0.41–1), and NPV of 0.85 (0.84–0.86). Cohen’s kappa for agreement of predicted and ground truth classes in the Würzburg sample was 0.2 (95% CI [0.1–0.3], simulated *p* = 0.0006). The performance of the XGB classifier remained relatively similar for the Würzburg sample (Cohen’s kappa 0.19 [0.09–0.28], *p* = 0.001).

The LR classifier trained on the Dalhousie University sample (45%/55% responders/non-responders) achieved a Cohen’s kappa of 0.15 (95% CI [0.07–0.24], simulated *p* = 0.0019), accuracy of 0.61 (95% CI [0.57–0.64]), AUC-ROC 0.62 (0.58–0.65), sensitivity 0.23 (0.16–0.31), specificity 0.91 (0.87–0.95), PPV of 0.68 (0.58–0.78), and NPV of 0.59 (0.57–0.62). However, the Cohen’s kappa obtained by the XGB classifier on the Dalhousie University sample was 0.1 (0.02–0.17), with a simulated *p* value of 0.03, suggesting this was more likely due to chance than the performance obtained with the LR classifier.

### Leave-one-site-out and predict-one-site-out analyses

Table 4 displays the results of the leave-one-site-out analysis using the LR classifier, and Table S2 in the Appendix displays the results using the XGB classifier. Removal of the Dalhousie University sample did not affect the classification performance (kappa 0.02 [0–0.04]), whereas classification performance remained better than chance, although quite weak, with removal of the Würzburg sample (kappa 0.05 [0.04–0.07], *p* = 0.003). Removal of data from San Diego and Barcelona result in Cohen’s kappa values of 0.06 (95% CI [0.04, 0.08], *p* = 0.002) and 0.05 ([0.04, 0.06], *p* = 0.005). Conversely, under the predict-one-site-out analysis, no combination of $n_{site}-1$ sites showed the ability to meaningfully predict samples from the left out site (Table S3 in Appendix).

**Table 4:** The results of the logistic regression (LR) classifier in the leave-one-site-out analyses.

Centre (LR+/LR−; %)	AUC	Accuracy	F1	NPV	PPV	Sensitivity	Specificity	Kappa
ALL (29/71)	0.57 (0.55, 0.6)	0.7 (0.7, 0.71)	0.08 (0.05, 0.1)	0.72 (0.71, 0.72)	0.36 (0.27, 0.46)	0.04 (0.03, 0.06)	0.97 (0.97, 0.98)	0.02 (− 0.01, 0.04)
Barcelona (27/73)	0.59 (0.58, 0.6)	0.72 (0.71, 0.72)	0.09 (0.07, 0.12)	0.72 (0.72, 0.72)	0.58 (0.53, 0.63)	0.05 (0.04, 0.07)	0.98 (0.98, 0.99)	0.05 (0.04, 0.06)^a^
Cagliari (28/72)	0.59 (0.57, 0.61)	0.72 (0.71, 0.73)	0.08 (0.03, 0.14)	0.72 (0.71, 0.73)	0.51 (0.22, 0.8)	0.04 (0.01, 0.08)	0.99 (0.98, 0.99)	0.05 (0.01, 0.09)
Geneva (23/77)	0.59 (0.57, 0.6)	0.71 (0.71, 0.72)	0.08 (0.07, 0.1)	0.72 (0.72, 0.72)	0.55 (0.47, 0.63)	0.05 (0.04, 0.05)	0.98 (0.98, 0.99)	0.04 (0.03, 0.05)
Halifax (45/55)	0.59 (0.56, 0.62)	0.75 (0.74, 0.75)	0.03 (0.01, 0.05)	0.75 (0.74, 0.75)	0.7 (0.31, 1)	0.01 (0, 0.03)	1 (1, 1)	0.02 (0, 0.04)
Japan (24/76)	0.59 (0.56, 0.62)	0.71 (0.71, 0.72)	0.09 (0.06, 0.12)	0.72 (0.71, 0.72)	0.54 (0.44, 0.63)	0.05 (0.03, 0.07)	0.98 (0.98, 0.99)	0.04 (0.02, 0.07)
Mayo (23/77)	0.58 (0.57, 0.6)	0.71 (0.71, 0.72)	0.1 (0.08, 0.11)	0.72 (0.72, 0.72)	0.51 (0.44, 0.59)	0.05 (0.04, 0.06)	0.98 (0.97, 0.99)	0.04 (0.03, 0.05)
Paris (18/82)	0.59 (0.57, 0.61)	0.7 (0.7, 0.71)	0.08 (0.06, 0.1)	0.71 (0.71, 0.71)	0.52 (0.43, 0.61)	0.04 (0.03, 0.05)	0.98 (0.98, 0.99)	0.03 (0.02, 0.05)
Poznan (48/52)	0.58 (0.57, 0.59)	0.73 (0.72, 0.73)	0.07 (0.04, 0.1)	0.73 (0.73, 0.73)	0.61 (0.51, 0.71)	0.04 (0.02, 0.05)	0.99 (0.99, 1)	0.04 (0.02, 0.06)
Romania (21/79)	0.6 (0.58, 0.62)	0.71 (0.7, 0.72)	0.1 (0.06, 0.14)	0.72 (0.71, 0.72)	0.59 (0.39, 0.8)	0.05 (0.03, 0.08)	0.99 (0.98, 0.99)	0.05 (0.02, 0.09)^a^
San Diego (11/89)	0.6 (0.59, 0.61)	0.7 (0.69, 0.7)	0.13 (0.1, 0.16)	0.7 (0.7, 0.71)	0.54 (0.46, 0.62)	0.07 (0.06, 0.09)	0.97 (0.96, 0.98)	0.06 (0.04, 0.08)a
Sweden (25/55)	0.54 (0.51, 0.57)	0.74 (0.74, 0.75)	0.05 (0.02, 0.08)	0.74 (0.74, 0.75)	0.55 (0.27, 0.82)	0.03 (0.01, 0.04)	1 (0.99, 1)	0.03 (0.01, 0.05)
Sydney (20/80)	0.59 (0.57, 0.61)	0.71 (0.71, 0.72)	0.08 (0.07, 0.09)	0.72 (0.71, 0.72)	0.52 (0.41, 0.63)	0.04 (0.04, 0.05)	0.98 (0.98, 0.99)	0.04 (0.02, 0.05)
Taiwan (14/86)	0.59 (0.58, 0.59)	0.71 (0.7, 0.71)	0.08 (0.06, 0.11)	0.71 (0.71, 0.72)	0.48 (0.39, 0.57)	0.05 (0.03, 0.06)	0.98 (0.97, 0.98)	0.04 (0.01, 0.06)
Würzburg (17/83)	0.6 (0.57, 0.62)	0.71 (0.7, 0.71)	0.11 (0.08, 0.14)	0.71 (0.71, 0.72)	0.55 (0.48, 0.63)	0.06 (0.04, 0.08)	0.98 (0.97, 0.99)	0.05 (0.04, 0.07)^a^

Table columns represent different classification statistics and values represent the mean of each statistic over five folds along with an empirical 95% confidence interval. The center column shows the center that was left out for each row. We have included the results of the aggregate analysis in the top row for ease of comparison. In the column listing respective centres, we have included the percentage of lithium responders (LR+) and non-responders (LR−) in the format %LR+/%LR− to provide context for the classification results. Asterisks signify that the given metric was found to have a *p* value less than 0.01 compared to the simulated null classifier. Abbreviations: area under the receiver operating characteristic curve (AUC), positive predictive value (PPV), negative predictive value (NPV), F-1 score (F1).

^a^Kappa value was found to have a *p* value less than 0.01 in comparison to the null classifier.

### Feature importance and gene set analysis

Rank correlations between the feature importance/coefficients are shown in Table 5. There was a statistically significant correlation in feature rankings between the XGBoost algorithm and the LR model when trained on the Dalhousie University (tau = 0.156, $\hbox {p}<0.001$) and Würzburg data (tau = 0.115, $\hbox {p}<0.001$) respectively. However, there was no evidence of correlation in feature importance/coefficients obtained with the same architectures trained on the Dalhousie and Würzburg sites, respectively (Dalhousie vs. Würzburg LR tau = − 0.003, *p* = 0.28; Dalhousie vs. Würzburg XGB tau = 0.007, *p* = 0.109).

**Table 5:** Feature importance comparisons. Logistic regression (LR) coefficients and XGBoost (XGB) feature importance were saved at each fold of cross-validation within the site-level analyses.

Comparison	Tau	*p* value
Dalhousie (LR) vs. Dalhousie (XGB)	0.156	< 0.001
Würzburg (LR) vs. Würzburg (XGB)	0.115	< 0.001
Dalhousie (LR) vs. Würzburg (LR)	− 0.003	0.280
Dalhousie (XGB) vs. Würzburg (XGB)	0.007	0.109

We computed the mean values of these coefficients (feature importances) over the cross-validation folds for each feature in the dataset (i.e. each locus of variation). We then compared the feature importance/coefficient values between models within a site (i.e. Dalhousie (LR) vs. Dalhousie (XGB)) and between sites within a model (i.e. Würzburg (LR) vs. Dalhousie (LR)). Where the comparison was made between the LR and XGB classifiers, LR coefficients were first transformed by taking their absolute value.

Tables 6 and S4 show the results of the gene ontology analyses using the 2,279 genes that overlapped between the Dalhousie and Würzburg site-level analyses with the LR classifier after quality control. The most overrepresented cellular component in our gene set was the postsynaptic membrane (88 genes, enrichment factor 1.71, *p* = 2.4e−05, and FDR 8.14e−03). A summary of the postsynaptic membrane genes is shown in Table S4. Results for the gene ontology analyses on the Dalhousie and Würzburg sites individually can be found in tables S5–S9 in the Appendix.

**Table 6:** Results from the GO cellular component analysis using genes selected by taking the same sign logistic regression coefficients for all SNPs in the Dalhousie and Würzburg samples that overlapped.

Functional class	N Ref	N Obs	N Exp	Factor	p (Raw)	FDR
Postsynaptic membrane (GO:0045211)	190	97	56.79	1.71	2.40E−05	8.14E−03
Synaptic membrane (GO:0097060)	252	124	75.32	1.65	6.28E−06	3.55E−03
Synapse (GO:0045202)	618	253	184.72	1.37	1.61E−05	6.83E−03
Synapse part (GO:0044456)	494	201	147.66	1.36	1.78E−04	3.78E−02
Cell junction (GO:0030054)	634	254	189.5	1.34	4.84E−05	1.17E−02
Neuron part (GO:0097458)	871	335	260.34	1.29	3.56E−05	1.01E−02
Cell projection (GO:0042995)	1055	387	315.34	1.23	2.47E−04	4.65E−02
Cell periphery (GO:0071944)	2461	866	735.59	1.18	4.15E−07	7.04E−04
Plasma membrane (GO:0005886)	2407	843	719.45	1.17	1.34E−06	1.14E−03

### Effects of follow-up method and narrowing definition thresholds

Table S10 in the Appendix shows that classification performance did not notably improve in any circumstance with narrowing of the classes to those subjects with more extreme Alda scores. Under the conditions of the most extreme narrowing (removal of Alda scores 4–8), we observed a complete decay to random chance in the classification performance in the Dalhousie and Würzburg samples with the LR classifier (kappa of 0.0 [0.0,0.0]).

Table S11 in the Appendix suggests that mode of patient follow-up (prospective or not) may have a small but above-chance effect on classification performance in these data. On the data pooled across sites employing prospective follow-up, the aggregate analysis achieved a kappa of 0.09 (0.04, 0.14), whereas in the non-prospective follow-up set, classification performance was effectively at the level of chance (kappa 0 [− 0.01, 0.01]).

In the LR model trained on the prospectively collected data, the most informative variants were associated with genes that showed statistically significant overrepresentation in synaptic membranes (both presynaptic [1.99-fold enrichment, FDR = 2.23e−02] and postsynaptic [1.75-fold enrichment, FDR = 9.37e−03] compartments; Table S12 in Appendix), with additional overrepresentation in several biological processes (most substantially neurogenesis [1.35-fold enrichment; FDR = 3.5e−02]). Further gene set analysis results are reported tables S12 - S14 in the Appendix.

## Discussion

To our knowledge, the present study is the first attempt to classify lithium response in an out-of sample fashion using genomic data alone: a task which our results suggest is an ongoing challenge. However, our results also suggest that this may be related to heterogeneity that arises from multi-site data pooling. In two subsamples of our genomic dataset, we found non-trivial classification performance informed by variants associated with a cellular component particularly relevant for the pathophysiology of BD. Furthermore, we observed non-trivial classification performance on data pooled across sites whose data were collected prospectively. These results suggest particular future directions that may be followed for the purpose of improving lithium response prediction models.

When trained on data pooled across all 14 sites, classification performance with the LR and XGBoost classifiers was effectively trivial, with Cohen’s kappa values of 0.02 (95% CI [− 0.01, 0.04]) and 0.0 (− 0.02, 0.03), respectively. However, the inability to classify lithium response was relatively consistent across analyses at the site-level, with the exception of data from Dalhousie University in the Canadian Maritimes (LR kappa 0.15 [0.07, 0.24], *p* = 0.002) and Würzburg, Germany (LR kappa 0.2 [0.1, 0.3], *p* = 0.0006). In both cases, the classifiers showed better specificity (LR Halifax 0.91 [0.87, 0.95]; LR Würzburg 1.0 [1.0, 1.0]) than sensitivity (LR Halifax 0.23 [0.16, 0.31]; LR Würzburg 0.13 [0.07, 0.02]), suggesting that improvements are needed for reducing the false negative rate. Classification performance remained trivial when data from the Halifax site was excluded (Cohen’s kappa 0.02 [0.0, 0.04]), likely due to poor sensitivity (0.01 [0, 0.03]). Interestingly, when the Würzburg dataset was left out of the aggregate analysis, the LR classifier’s performance increased to a small but above chance level (0.05 [0.04, 0.07], *p* = 0.003), which was largely driven by an improvement in PPV (from 0.36 [0.27, 0.46] to 0.55 [0.48–0.63]), without loss of NPV. The low performance in the aggregate analysis combined with variability in the class imbalance and site-level classification performance indicate that there may be a non-trivial level of between-site heterogeneity in our data, and this may be negatively impacting classification performance. It is also possible that there exists significant between-site heterogeneity in clinical phenotype—as recently demonstrated by Nunes et al.^10^—which could also negatively impact classification performance. Future research must investigate the relationship between heterogeneity in clinical phenotype and classification performance in multi-site studies.

Although there was no statistically significant rank correlation between the most important individual features from the LR classifiers trained on the Halifax and Würzburg samples, respectively, there were interesting patterns of gene enrichment among the specific variants whose direction of effect was consistent between these sites. Specifically, we noted the greatest enrichment among genes associated with the postsynaptic membrane (1.71-Fold enrichment; FDR = 8.14e−03). The overrepresented gene set included genes such as ANK3, DISC1, various glutamate receptor genes, scaffolding-related genes such as HOMER1, and adhesion-molecule related genes such as the cadherin (CDH) family. Postsynaptic membrane function, and several of the enriched genes above, have previously been associated with BD. For example, ankyrin 3 (ANK3), which is involved in the aggregation of voltage-gated sodium channels, has been associated with BD in multiple studies^11–13^ and may be downregulated by lithium^14^. Pickard et al.^15,16^ demonstrated association between BD and kainate receptor subunit genes (namely KA1 [GRIK4]) which have a significant role in regulation of cellular excitability^17^. The DISC1 gene, which encodes an integral postsynaptic regulator of plasticity^18^, was notably associated with schizophrenia and affective disorders in a Scottish family study^19,20^. However, lithium response more specifically may have narrower associations with postsynaptic elements involved in G-protein coupled receptor signaling pathways^21–23^. This pathway was not statistically overrepresented in gene set overlapping between the Würzburg and Halifax datasets. Since genetic variants’ LR coefficient ranks were uncorrelated between these sites— suggesting a degree of between-site heterogeneity in feature importance—it may consequently be easier to find enrichment of genes in more general biological processes/components. Indeed, when we repeated the aggregate analysis by restricting data to only those collected in a prospective fashion (thereby removing some heterogeneity due to methodological differences), we found further overrepresentation of genes involved in some biological processes (especially neurogenesis [1.35-fold; FDR = 3.50e−02]). A plausible hypothesis is that (A) informative variants from models trained on substantially heterogeneous data will associate with relatively broad and non-specific biological pathways, and that (B) the associated biological pathways may increase in specificity for models trained on more homogeneous datasets. Testing this hypothesis will require further work toward identifying potentially more homogeneous subgroups within each of the lithium responder and non-responder classes.

Schnack and Khan^24^ opined that between-site heterogeneity could reduce classification performance in multi-site ML studies, supporting their argument with results aggregated from ML studies in schizophrenia neuroimaging. They suggested that small studies are more likely to consist of homogeneous and biased samples, but that increasing sample size entails a commensurate increase in heterogeneity. If sampling biases are related to the site from which data are sourced, then increasing sample size by way of pooling data in multi-site studies should consequently result in a more heterogeneous dataset for which classification performance degrades. However, we have previously found contradictory evidence in both BD neuroimaging and in prediction of lithium response, where pooling data across sites improved classification performance^10,25^. In both of those studies, however, there was a relatively strict set of harmonization procedures; the former study employed the harmonized data-handling procedures of the ENIGMA consortium (see the appendix of Nunes et al.^25^ for details), while the latter included only subjects that were followed prospectively for at least one year before assessment of lithium responsiveness using the Alda scale. It is possible that this standardization may have reduced the degree of between-site heterogeneity in those data, thereby allowing phenotypic heterogeneity to be the primary source of variation in their data.

Data collection practices between sites in our study may have influenced classification performance. A LR classifier applied to the aggregated dataset from only those sites employing prospective follow up achieved superior performance (kappa 0.09 [0.04, 0.14]) in comparison to classification on aggregated data from the non-prospective sites (kappa 0.0 [− 0.01, 0.01]; Table S10, Appendix), although classification performance remains weak overall. This may have been due to the reduced sample size in each aggregate analysis. Importantly, however, between-site heterogeneity due to data collection practices was likely more influential on classification performance than heterogeneity in lithium responsiveness proper, since repetition of the classification analyses with progressively narrowed Alda score thresholds did not change classification performance (Table S9 in Appendix). In other words, we failed to demonstrate stronger genetic separability among subjects with the most extreme (high or low) Alda scores. Hypothetically, these should be the most “clear” responders and non-responders. Together, these results suggest that subjects who are prospectively followed are likely better “exemplars” of lithium responsiveness, but that this representativeness is not well captured by the degree of extremeness in the Alda score. This leaves open the possibility of other dimensions along which exemplars of lithium responsiveness/non-responsiveness could be identified. For instance, lithium non-responders in the present study showed a greater propensity for psychosis at baseline (721/1576 [45.7%]) compared to lithium responders (253/634 [40%], *p* = 0.001). Clinical features such as history of psychosis, clinical course, and history of rapid cycling may indeed represent feature dimensions along which we may identify clinical exemplars of lithium responsiveness and non-responsiveness, respectively^10,26–28^. Future studies should therefore (A) maximize inclusion of subjects who are prospectively followed, and (B) investigate alternative methods for identifying exemplars of lithium responsiveness.

One limitation of the present study is the use of only SNPs that overlapped across all genotyping platforms in the dataset. This may have biased the available feature set, and had lower overall genome coverage. However, there were no large gaps in genome coverage when using only this restricted set of SNPs. Furthermore, use of the fully imputed data would have been infeasible without some degree of feature pre-selection, for which optimal performance in high-dimensional feature spaces can be intractable. Moreover, imputation will introduce additional assumptions and nevertheless also depends in some way on the observable data. For this reason, we opted to not replace directly genotyped features with imputed ones. Given that nearly 50,000 features were directly genotyped in all subjects, of which there were only 2210, we opted to not increase the feature space dimensionality by adding imputed variants to our model. Our analysis was based on SNPs and thus it does not account for changes in gene expression. This could be another possible explanation of differences between responders and non-responders as shown by Hunsberger et al.^29^ who found a differential pattern of mi-RNA $\times$ mRNA interactions between the two groups of patients in an in vitro study.

Another limitation of our study is that hyperparameter tuning was not performed on the models implemented. This was avoided since appropriately guarding against overfitting would necessitate a nesting of cross-validation procedures. Nested cross-validation requires larger sample sizes, and some of the sites in our data had relatively few subjects. As a result, significant inequality between sites would exist in terms of the benefits of hyperparameter optimization. Comparability of performance in the site-level analyses, and the inference of more general conclusions, would therefore have been more challenging if hyperparameter optimization was used. Furthermore, for the logistic regression analysis, the $L_2$ regularization was set with a constant strength of $C=1$, which corresponds to a standard normal prior on the regression weights. This consistent prior on the regression weights was necessary in order for them to be comparable across cross-validation folds, and thus to be used for the pathway analysis. Hyperparameter optimization would have yielded weights that came from different priors and complicated such a comparison.

In conclusion, the present study suggests promise for the out-of-sample genomic classification of lithium response, but highlights the ongoing challenges of this task. Future modeling decisions should include not only the goal of achieving above-chance omnibus classification performance, but also a robust sensitivity. Several concrete steps in this direction include increasing sample sizes and further harmonizing data collection procedures.

## Supplementary Information

Supplementary Information.

## Data Availability

This paper is based on data obtained by the ConLiGen consortium and previously published (Hou et al. Lancet 2016). Interested investigators are able to request the data from the Consortium (http://www.conligen.org/) upon submitting a proposal for secondary analysis. Dr. M. Alda, the corresponding author on this paper, can be contacted for further instructions.
