# Supplementary material for: Prediction of lithium response using genomic data
Source: Sci Rep. 2021 Jan 13;11:1155. doi: 10.1038/s41598-020-80814-z (PMC7806976; doi:10.1038/s41598-020-80814-z)
Supplement: Supplementary file 1 — Supplementary Information. [file 41598_2020_80814_MOESM1_ESM.pdf]

Supplementary Materials

Prediction of Lithium Response Using Genomic Data

William Stone, Abraham Nunes, Kazufumi Akiyama, Nirmala Akula, Raffaella Ardau, Jean-Michel Aubry, Lena Backlund, Michael Bauer, Frank Bellivier, Pablo Cervantes, Hsi-Chung Chen, Caterina Chillotti, Cristiana Cruceanu, Alexandre Dayer, Franziska Degenhardt, Maria Del Zompo, Andreas J. Forstner, Mark Frye, Janice M. Fullerton, Maria Grigoroiu-Serbanescu, Paul Grof, Ryota Hashimoto, Liping Hou, Esther Jiménez, Tadafumi Kato, John Kelsoe, Sarah Kittel-Schneider, Po-Hsiu Kuo, Ichiro Kusumi, Catharina Lavebratt, Mirko Manchia, Lina Martinsson, Manuel Mattheisen, Francis J. McMahon, Vincent Millischer, Philip B. Mitchell, Markus M. Nothen, Claire O'Donovan, Norio Ozaki, Claudia Pisanu, Andreas Reif, Marcella Rietschel, Guy Rouleau, Janusz Rybakowski, Martin Schalling, Peter R. Schofield, Thomas G. Schulze, Giovanni Severino, Alessio Squassina, Julia Veeh, Eduard Vieta, Thomas Trappenberg, Martin Alda

CONTENTS

Additional Details on Data Collection 2
Simulated p-Values of Null Classifiers 3
Additional analysis results 5
Aggregate and Site level Analysis 5
Table S1 5
Leave One Site Out Analysis 6
Table S2 6
Predict One Site Out Analysis 7
Table S3 7
Analysis under 100-Fold Shuffle-Split Partitioning 8
Gene Set Analyses in the Halifax and Würzburg Samples 11
Table S4 11
Table S5 14
Table S6 16
Table S7 20
Table S8 27
Table S9 28
Classification Analysis with Progressively Narrowed Lithium Response Definitions 33
Table S10 33

Classification Analysis Stratified by Follow-Up Methodology 41
Classification Results 41
Table S11 41
Gene Set Analyses 44
Table S12 44
Table S13 46
Table S14 48
Principal Component Analysis 57
References 59

Additional Details on Data Collection

Prior to the original study, each data collection center acquired ethical approval. All participants provided written informed consent, and their data were anonymized. Patients with DSM-III or DSM-IV diagnosis of bipolar I or bipolar II disorder and treated with lithium as their only mood stabilizer for a minimum of 6 months were included in the study. The response to lithium has been quantified using the "Retrospective Criteria of Long-Term Treatment Response in Research Subjects with Bipolar Disorder'' (1). The scale quantifies change of the frequency, duration and severity of episodes during long term on a 0 to 10 scale (score A) and corrects it for five criteria (B1 – B5), each scored 0, 1 or 2, that reflect possible confounding factors. The total score is then derived by subtracting the sum of the B scores from the A score. The total score is truncated at 0 so that the range of the total scores is between 0 and 10. Scores of 7 and higher indicate good and scores of 6 or lower poor response to treatment. The investigators were trained in the use of the scale and obtained adequate inter-rater reliability (2).

DNA was extracted from peripheral blood samples. Samples were genotyped at the NIMH, University of Bonn, or Broad Institute using either Affymetrix or Illumina SNP arrays according to the manufacturers' protocols. Quality control and imputation were carried out in batches based on the genotyping arrays and origin of the samples. As described, only SNPs common to all genotyping platforms were used in our analysis.

The following quality control parameters were applied: per subject genotype missingness < 0.03; autosomal heterozygosity rate within mean±3SD; MAF ≥ 0.01; SNP missingness < 0.05; and SNP Hardy-Weinberg equilibrium $p > 10^{-4}$ in all samples; no discrepancies were detected between reported sex and sex as indicated by genotypes on chromosome X. After basic quality control and linkage disequilibrium (LD) pruning, about 40K directly genotyped SNPs shared across all SNP arrays were used for the relatedness testing by PLINK. Only autosomal SNPs with a MAF ≥ 0.05 were used for these analyses. Duplicated samples and cryptically related pairs (Pi_hat > 0.10) were identified; only one member of each relative pair was kept for the data analyses. EIGENSOFT was used to identify population outliers, which were removed.

Simulated p-Values of Null Classifiers

We computed p-values for the classification performance measures by simulating the performance of a null classifier. Here, we describe this procedure formally, although the procedure is implemented in Python 3.5+ code found at (https://github.com/abrahamnunes/criticism).

We are given a dataset $\mathcal{D} = (\mathbf{x}_i, y_i)_{i=1}^{n_s}$ with n_x-dimensional feature vectors $\mathbf{x}_i$ and binary class labels y_i for n_s subjects indexed by $i \in \{1, 2, \ldots, n_s\}$. During model criticism, the dataset is split into disjoint training and validation partitions

$$\mathcal{D} = \left\{\mathcal{D}_T^{(k)}, \mathcal{D}_V^{(k)}\right\}_{k=1}^{K},$$

where the subscripts T and V denote the training and validation sets, respectively, and the cross-validation folds are indexed by $k \in \{1, 2, \ldots, K\}$. Importantly, the cross-validation partitions are done in a *stratified* fashion, such that the probability of $y_i = 1$ (in our study this would be the probability of lithium response) is approximately equal in both the training and validation sets.

Under the use of stratified cross-validation, we can compute the baseline probability of the positive class as

$$\alpha = \frac{1}{n_s} \sum_{i=1}^{n_s} \mathbb{I}[y_i = 1],$$

where $\mathbb{I}[\cdot]$ is an indicator function that evaluates to 1 if its argument is true. Now let β represent the classifier's sensitivity, and γ its specificity. Given only α and the number of subjects n_s, we can specify prior distributions on β and γ of a "null classifier" as follows:

$$\beta \sim \mathrm{Beta}(\frac{1}{2}\alpha n_s, \frac{1}{2}(1-\alpha)n_s)$$

$$\gamma \sim \mathrm{Beta}(\frac{1}{2}(1-\alpha)n_s, \frac{1}{2}\alpha n_s)$$

We note that a classifier with sensitivity and specificity sampled from these distributions is trivial if we recall that the parameters of a beta distribution are pseudocounts. Since the first argument in each of the Beta(., .) functions is the pseudocount for the positive class, we can see that, given a sample of size n_s, the expected number of times that a null classifier will identify a subject as being a lithium responder when he or she truly is one will be $\alpha n_s/2$, and the expected number of times that the null classifier will identify a lithium responder as a non-responder is $(1-\alpha)n_s/2$. We can interpret the parameters of the prior on γ in the same way, except reversed in order.

Given β and γ, we can compute the null classifier's expected confusion matrix for the sample of size n_s. If y denotes the true label and $\hat{y}$ denotes the predicted label, we have the following confusion matrix.

	$\hat{y} = 0$	$\hat{y} = 1$
$y = 0$	$(1-\alpha)\gamma$	$(1-\alpha)(1-\beta)$
$y = 1$	$\alpha(1-\beta)$	$\alpha\beta$

which corresponds to

	$\hat{y} = 0$	$\hat{y} = 1$
$y = 0$	P_{TN}	P_{FP}
$y = 1$	P_{FN}	P_{TP}

and from which we can compute the Cohen's kappa metric as follows:

- $PChance(\alpha, \beta, \gamma) = (P_{TP} + P_{FN})\,(P_{TP} + P_{FP}) + (P_{TN} + P_{FN})\,(P_{TN} + P_{FP})$
- $Kappa(\alpha, \beta, \gamma) = \dfrac{P_{TP} + P_{TN} - PChance(\alpha, \beta, \gamma)}{1 - PChance(\alpha, \beta, \gamma)}$

Let the null classification performance statistic be denoted as τ, and let t^* denote the performance statistic computed for the actual model predictions $\hat{y}$ and $\hat{p}$ (where the latter represent the predicted probabilities of the positive class by the trained classifier $\mathcal{M}$). The simulated probability that the null classifier obtains performance greater than $\mathcal{M}$ under the given performance metric is $p(\tau > t^*)$. Given the M simulations of the null classifier's performance, we can approximate this tail probability as

$$p = \frac{1}{M} \sum_{i=1}^{M} \mathbb{I}[\tau_i > t^*]$$

where τ_i is the i'th sample of the null classifier's performance under a binary response set of size n_s.

Default parameters were used in all analysis that involved using the criticism package.

Additional analysis results

Aggregate and Site level Analysis

Table S1

Table S1: The performance of the xgboost (XGB) classifier in the aggregate and site-level analyses. Table columns represent different classification statistics and values represent the mean of each statistic over five folds along with an empirical 95% confidence interval. Asterisks signify that the given metric was found to have a p-value less than 0.01 in comparison to the simulated null classifier. *Abbreviations*: all sites (ALL; i.e. aggregate analysis), area under the receiver operating characteristic curve (AUC), positive predictive value (PPV), negative predictive value (NPV), F-1 score (F1).

Centre	AUC	Accuracy	F1	NPV	PPV	Sensitivity	Specificity	Kappa
ALL	0.57 (0.55, 0.6)	0.7 (0.7, 0.71)	0.05 (0.02, 0.07)	0.71 (0.71, 0.72)	0.3 (0.14, 0.45)	0.03 (0.01, 0.04)	0.98 (0.97, 0.98)	0.0 (-0.02, 0.03)
Barcelona	0.52 (0.42, 0.61)	0.69 (0.65, 0.73)	0.07 (0.0, 0.2)	0.72 (0.7, 0.75)	0.1 (0.0, 0.3)	0.05 (0.0, 0.15)	0.93 (0.89, 0.96)	-0.04 (-0.16, 0.08)
Cagliari	0.53 (0.46, 0.59)	0.71 (0.7, 0.73)	0.03 (0.0, 0.08)	0.72 (0.72, 0.72)	0.07 (0.0, 0.2)	0.02 (0.0, 0.05)	0.99 (0.96, 1.0)	0.0 (-0.0, 0.01)
Geneva	0.8 (0.65, 0.94)	0.74 (0.68, 0.8)	0.0 (0.0, 0.0)	0.76 (0.73, 0.8)	0.0 (0.0, 0.0)	0.0 (0.0, 0.0)	0.96 (0.9, 1.0)	-0.06 (-0.13, 0.01)
Halifax	0.57 (0.52, 0.63)	0.56 (0.52, 0.6)	0.45 (0.4, 0.5)	0.59 (0.56, 0.61)	0.52 (0.46, 0.58)	0.41 (0.33, 0.49)	0.69 (0.59, 0.78)	0.1 (0.02, 0.17)
Japan	0.42 (0.33, 0.51)	0.73 (0.71, 0.76)	0.0 (0.0, 0.0)	0.75 (0.74, 0.76)	0.0 (0.0, 0.0)	0.0 (0.0, 0.0)	0.97 (0.94, 0.99)	-0.04 (-0.08, -0.01)
Mayo	0.71 (0.56, 0.85)	0.78 (0.76, 0.79)	0.07 (0.0, 0.2)	0.77 (0.76, 0.79)	0.2 (0.0, 0.59)	0.04 (0.0, 0.12)	1.0 (1.0, 1.0)	0.05 (-0.05, 0.16)
Paris	0.52 (0.43, 0.6)	0.82 (0.81, 0.83)	0.0 (0.0, 0.0)	0.82 (0.81, 0.83)	0.0 (0.0, 0.0)	0.0 (0.0, 0.0)	1.0 (1.0, 1.0)	0.0 (0.0, 0.0)
Poznan	0.6 (0.5, 0.7)	0.61 (0.5, 0.72)	0.55 (0.41, 0.69)	0.61 (0.51, 0.72)	0.62 (0.5, 0.75)	0.51 (0.34, 0.69)	0.7 (0.58, 0.8)	0.21 (0.0, 0.43)
Romania	0.47 (0.35, 0.6)	0.79 (0.78, 0.8)	0.0 (0.0, 0.0)	0.79 (0.78, 0.8)	0.0 (0.0, 0.0)	0.0 (0.0, 0.0)	1.0 (1.0, 1.0)	0.0 (0.0, 0.0)
San Diego	0.44 (0.38, 0.5)	0.89 (0.88, 0.9)	0.0 (0.0, 0.0)	0.89 (0.88, 0.9)	0.0 (0.0, 0.0)	0.0 (0.0, 0.0)	1.0 (1.0, 1.0)	0.0 (0.0, 0.0)
Sweden	0.52 (0.48, 0.56)	0.52 (0.47, 0.57)	0.37 (0.27, 0.48)	0.55 (0.51, 0.6)	0.45 (0.37, 0.52)	0.33 (0.22, 0.44)	0.68 (0.63, 0.73)	0.01 (-0.1, 0.12)
Sydney	0.41 (0.22, 0.59)	0.78 (0.73, 0.83)	0.0 (0.0, 0.0)	0.79 (0.76, 0.83)	0.0 (0.0, 0.0)	0.0 (0.0, 0.0)	0.98 (0.94, 1.0)	-0.03 (-0.08, 0.03)
Taiwan	0.54 (0.33, 0.75)	0.86 (0.84, 0.88)	0.0 (0.0, 0.0)	0.86 (0.84, 0.88)	0.0 (0.0, 0.0)	0.0 (0.0, 0.0)	1.0 (1.0, 1.0)	0.0 (0.0, 0.0)

Würzburg	0.6 (0.56, 0.65)	0.85 (0.83, 0.86)	0.22 (0.11, 0.33)	0.85 (0.84, 0.86)	0.7 (0.31, 1.0)	0.13 (0.07, 0.2)	0.99 (0.98, 1.0)	0.19 (0.09, 0.28)[a]
a - Kappa value was found to have a p-value less than 0.01 in comparison to the null classifier.								

Leave One Site Out Analysis

Table S2

Table S2: The results of the XGBoost classifier in the leave one site out analyses. Table columns represent different classification statistics and values represent the mean of each statistic over five folds along with an empirical 95% confidence interval. The center column shows the center that was left out for each row. Asterisks signify that the given metric was found to have a p-value less than 0.01 in comparison to the simulated null classifier. *Abbreviations*: area under the receiver operating characteristic curve (AUC), positive predictive value (PPV), negative predictive value (NPV), F-1 score (F1).

Centre	AUC	Accuracy	F1	NPV	PPV	Sensitivity	Specificity	Kappa
Barcelona	0.56 (0.55, 0.56)	0.71 (0.7, 0.71)	0.05 (0.02, 0.09)	0.71 (0.71, 0.72)	0.29 (0.11, 0.46)	0.03 (0.01, 0.05)	0.98 (0.97, 0.99)	0.01 (-0.01, 0.03)
Cagliari	0.56 (0.54, 0.57)	0.7 (0.69, 0.7)	0.05 (0.03, 0.08)	0.71 (0.71, 0.71)	0.28 (0.22, 0.33)	0.03 (0.01, 0.05)	0.97 (0.96, 0.98)	0 (-0.01, 0.01)
Geneva	0.55 (0.52, 0.59)	0.71 (0.7, 0.72)	0.07 (0.04, 0.1)	0.72 (0.71, 0.72)	0.43 (0.29, 0.58)	0.04 (0.02, 0.05)	0.98 (0.97, 0.99)	0.03 (0, 0.05)
Halifax	0.55 (0.52, 0.58)	0.75 (0.74, 0.75)	0.03 (0.02, 0.04)	0.75 (0.75, 0.75)	0.63 (0.42, 0.85)	0.02 (0.01, 0.02)	1 (0.99, 1)	0.02 (0.01, 0.03)
Japan	0.58 (0.55, 0.6)	0.71 (0.7, 0.71)	0.07 (0.03, 0.1)	0.71 (0.71, 0.72)	0.42 (0.28, 0.57)	0.04 (0.02, 0.05)	0.98 (0.98, 0.99)	0.02 (0, 0.05)
Mayo	0.57 (0.54, 0.59)	0.71 (0.7, 0.72)	0.07 (0.02, 0.12)	0.72 (0.71, 0.72)	0.42 (0.2, 0.63)	0.04 (0.01, 0.07)	0.98 (0.97, 0.99)	0.03 (-0.01, 0.07)
Paris	0.55 (0.53, 0.57)	0.69 (0.69, 0.7)	0.08 (0.05, 0.1)	0.7 (0.7, 0.71)	0.37 (0.24, 0.5)	0.04 (0.03, 0.06)	0.97 (0.97, 0.98)	0.02 (-0.01, 0.04)
Poznan	0.54 (0.53, 0.56)	0.72 (0.72, 0.72)	0.06 (0.05, 0.07)	0.73 (0.72, 0.73)	0.47 (0.4, 0.54)	0.03 (0.03, 0.04)	0.98 (0.98, 0.99)	0.03 (0.02, 0.03)
Romania	0.56 (0.53, 0.6)	0.7 (0.7, 0.71)	0.06 (0.04, 0.08)	0.71 (0.71, 0.71)	0.45 (0.33, 0.57)	0.03 (0.02, 0.04)	0.98 (0.97, 0.99)	0.02 (0, 0.04)
San Diego	0.58 (0.55, 0.61)	0.69 (0.68, 0.7)	0.09 (0.06, 0.12)	0.7 (0.7, 0.7)	0.47 (0.35, 0.59)	0.05 (0.03, 0.07)	0.97 (0.97, 0.98)	0.03 (0.01, 0.06)
Sweden	0.57 (0.55, 0.6)	0.74 (0.73, 0.75)	0.06 (0.03, 0.1)	0.74 (0.74, 0.75)	0.54 (0.24, 0.83)	0.03 (0.01, 0.05)	0.99 (0.98, 1)	0.03 (0, 0.06)
Sydney	0.54 (0.51, 0.56)	0.71 (0.7, 0.71)	0.07 (0.04, 0.1)	0.71 (0.71, 0.72)	0.42 (0.3, 0.53)	0.04 (0.02, 0.05)	0.98 (0.97, 0.99)	0.02 (0, 0.05)
Taiwan	0.54 (0.53, 0.55)	0.7 (0.7, 0.71)	0.06 (0.04, 0.08)	0.71 (0.71, 0.71)	0.44 (0.31, 0.58)	0.03 (0.02, 0.04)	0.98 (0.98, 0.99)	0.02 (0, 0.04)

Würzburg	0.57 (0.53, 0.6)	0.7 (0.69, 0.7)	0.07 (0.04, 0.09)	0.71 (0.7, 0.71)	0.39 (0.27, 0.5)	0.04 (0.02, 0.05)	0.97 (0.97, 0.98)	0.02 (-0.01, 0.04)

Predict One Site Out Analysis

Table S3

Table S3: Performance of the XGBoost classifier in the predict one site out analysis. Columns represent different classification metrics. The center shown in each row represents the center that the classifier was evaluated on, and was thus not present in the training set. *Abbreviations*: logistic regression (LR), xgboost classifier (XGB), area under the receiver operating characteristic curve (AUC), positive predictive value (PPV), negative predictive value (NPV), F-1 score (F1).

Model	Centre	AUC	Accuracy	F1	NPV	PPV	Sensitivity	Specificity	Kappa
LR	Barcelona	0.56	0.73	0	0.73	0	0	1.0	0
LR	Cagliari	0.6	0.72	0	0.72	0	0	1.0	0
LR	Geneva	0.53	0.77	0.13	0.78	0.5	0.08	0.98	0.08
LR	Halifax	0.56	0.55	0.01	0.55	0.5	0.01	0.99	0
LR	Japan	0.52	0.76	0	0.76	0	0	1.0	0
LR	Mayo	0.58	0.79	0.17	0.78	1.0	0.09	1.0	0.13
LR	Paris	0.5	0.82	0.05	0.82	1.0	0.03	1.0	0.04
LR	Poznan	0.59	0.52	0.04	0.52	0.5	0.02	0.98	0
LR	Romania	0.53	0.78	0.11	0.8	0.4	0.06	0.97	0.05
LR	San Diego	0.5	0.87	0	0.89	0	0	0.97	-0.05
LR	Sweden	0.57	0.57	0.08	0.56	1.0	0.04	1.0	0.05
LR	Sydney	0.41	0.79	0	0.79	0	0	1.0	0
LR	Taiwan	0.35	0.86	0	0.86	0	0	1.0	0
LR	Würzburg	0.44	0.79	0.05	0.83	0.12	0.03	0.95	-0.02
XGB	Barcelona	0.56	0.73	0	0.73	0	0	1.0	0
XGB	Cagliari	0.42	0.71	0	0.72	0	0	0.99	-0.01
XGB	Geneva	0.53	0.74	0	0.76	0	0	0.95	-0.06
XGB	Halifax	0.49	0.55	0.01	0.55	1.0	0.01	1.0	0.01
XGB	Japan	0.6	0.76	0	0.76	0	0	1.0	0
XGB	Mayo	0.51	0.76	0	0.76	0	0	0.99	-0.02

XGB	Paris	0.5	0.83	0.15	0.83	1.0	0.08	1.0	0.12
XGB	Poznan	0.49	0.49	0.04	0.51	0.25	0.02	0.94	-0.04
XGB	Romania	0.51	0.78	0.11	0.79	0.33	0.06	0.97	0.04
XGB	San Diego	0.5	0.87	0.06	0.89	0.12	0.04	0.96	0.01
XGB	Sweden	0.5	0.55	0.03	0.55	0.67	0.01	0.99	0.01
XGB	Sydney	0.43	0.78	0	0.79	0	0	0.98	-0.03
XGB	Taiwan	0.46	0.85	0	0.86	0	0	0.99	-0.02
XGB	Würzburg	0.54	0.83	0.12	0.84	0.5	0.07	0.99	0.08

Analysis under 100-Fold Shuffle-Split Partitioning

To evaluate the sensitivity of the primary analysis to the specific partitioning scheme used, we repeated the classification study using 100-fold stratified shuffle-split partitioning (often known as *Monte-Carlo Cross-Validation*). We evaluate this only for the logistic regression classifier, since this was our primary model architecture of interest (the XGBoost model was itself a measure of result sensitivity to model architectures that could capture nonlinearity). Figures S1 and S2 show the results of this analysis.

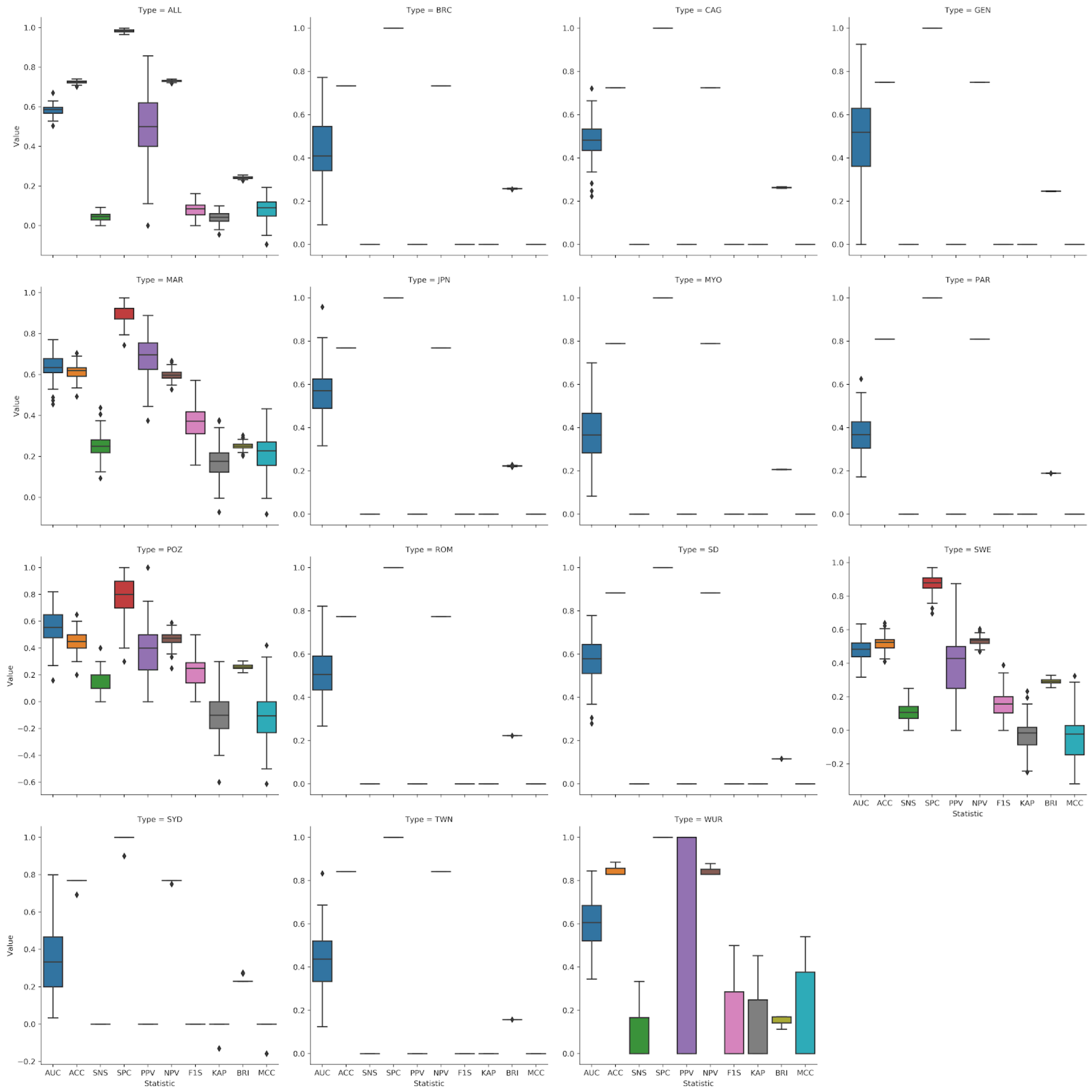

Figure S1: Results of re-analysis under 100-fold stratified shuffle-split train-test partitioning. This was repeated once for the aggregate dataset (ALL), and the site-level data. For each of 100 iterations, the data were randomly partitioned into training (80% of examples) and testing (20% of examples) sets. This evaluates the degree to which train/test composition in the original 5-fold cross-validation affected the results. Each panel represents the results for a given site in the data. Specific statistics are shown on the X-axes, and the statistic values are plotted on the y-axes. *Abbreviations:* area under the receiver operating characteristic curve (ALL), accuracy (ACC), sensitivity (SNS), specificity (SPC), positive predictive value (PPV), negative predictive value (NPV), F-1 Score (F1S), Cohen's kappa (KAP), Brier score (BRI), and Matthews correlation coefficient (MCC). *Site Abbreviations:* all sites (ALL), Barcelona (BRC), Cagliari (CAG), Geneva (GEN),

Halifax (Maritimes; MAR), Japan (JPN), Mayo clinic (MYO), Paris (PAR), Poznan (POZ), Romania (ROM), San Diego (SD), Sweden (SWE), Sydney (SYD), Taiwan (TWN), Wuerzburg (WUR).

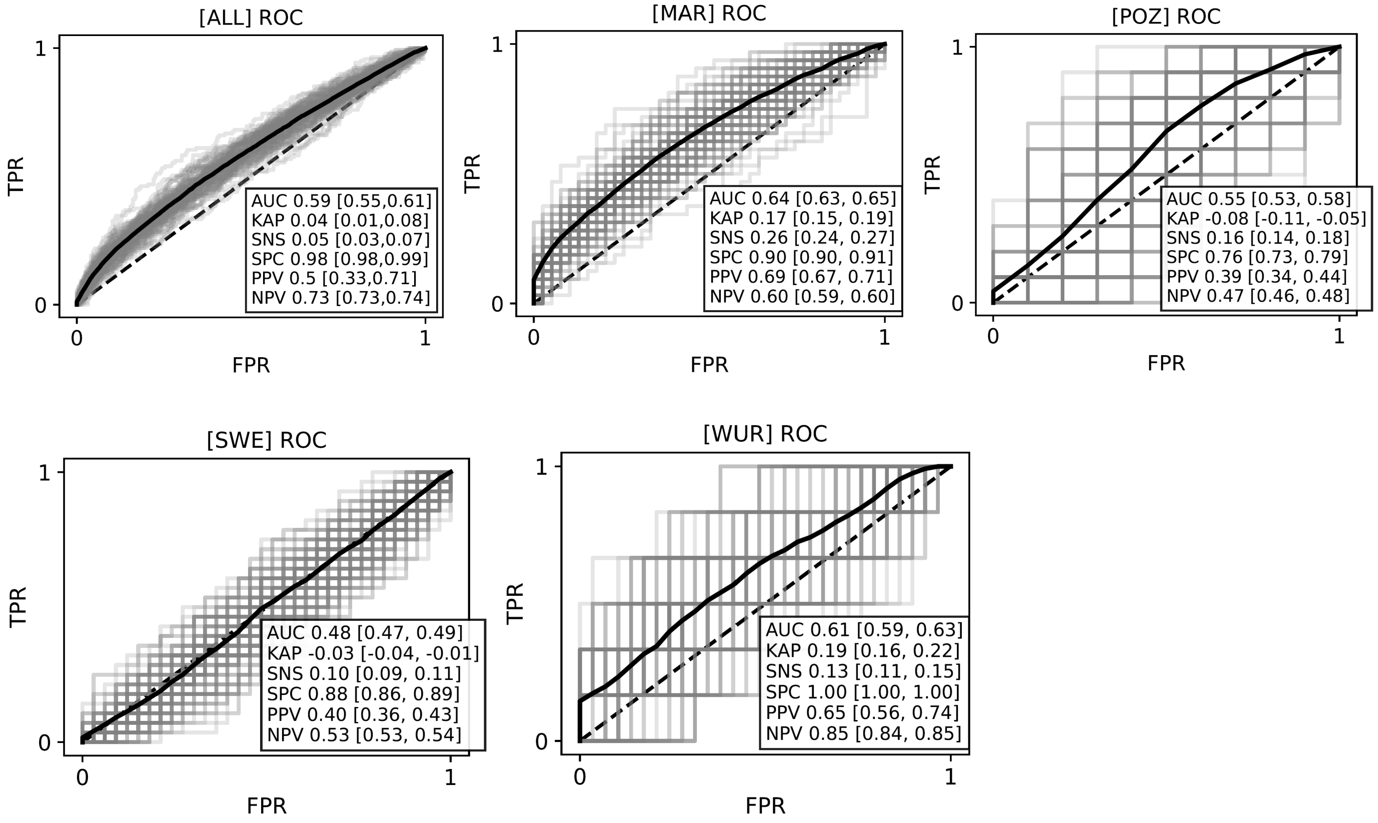

Figure S2: Receiver operating characteristic (ROC) curves from the aggregate dataset (ALL) as well as sites that achieved non-trivial performance (i.e. where positive and negative predictive values and Kappa scores were not fixed to 0 or 1, which indicates that these datasets did not result in classifiers that merely predicted all test subjects as part of the majority class). Dashed lines indicate chance performance. Average ROC curves are shown in black. Transparent gray curves are the ROC curves for individual folds of the 100-fold stratified shuffle-split procedure. *Abbreviations:* area under the curve (AUC), Cohen's kappa (KAP), sensitivity (SNS), specificity (SPC), positive and negative predictive values (PPV and NPV, respectively), all subjects (ALL), Halifax (Maritimes; MAR), Poznan (POZ), Sweden (SWE).

Gene Set Analyses in the Halifax and Würzburg Samples

Table S4

Table S4: Specific genes tagged with the ontology class "postsynaptic membrane" that were overrepresented in our gene set analysis.					
Gene ID	Gene	PANTHER Protein Class	Gene ID	Gene	PANTHER Protein Class
LRFN2	Leucine-rich repeat and fibronectin type-III domain-containing protein 2		*ANK3*	Ankyrin-3	
RGS9	Regulator of G-protein signaling 9		*PTPRT*	Receptor-type tyrosine-protein phosphatase T	protein phosphatase(PC00195);receptor(PC00197)
DLG1	Disks large homolog 1	transmembrane receptor regulatory/adaptor protein(PC00226)	*ADCY8*	Adenylate cyclase type 8	
CTNNA2	Catenin alpha-2	cell adhesion molecule(PC00069);non-motor actin binding protein(PC00165)	*SEMA4F*	Semaphorin-4F	membrane-bound signaling molecule(PC00152)
GRIK1	Glutamate receptor ionotropic, kainate 1		*GABRR1*	Gamma-aminobutyric acid receptor subunit rho-1	GABA receptor(PC00023);acetylcholine receptor(PC00037)
CADPS2	Calcium-dependent secretion activator 2	calcium-binding protein(PC00060)	*LRRC4C*	Leucine-rich repeat-containing protein 4C	
GRIP2	Glutamate receptor-interacting protein 2		*SLC6A11*	Sodium- and chloride-dependent GABA transporter 3	cation transporter(PC00068)
NTRK3	NT-3 growth factor receptor		*ADGRB1*	Adhesion G protein-coupled receptor B1	G-protein coupled receptor(PC00021);antibacterial response protein(PC00051);protease(PC00190)
DCC	Netrin receptor DCC		*SHISA6*	Protein shisa-6	
CDH9	Cadherin-9		*SLC8A3*	Sodium/calcium exchanger 3	
CHRM3	Muscarinic acetylcholine receptor M3	G-protein coupled receptor(PC00021)	*KCNH1*	Potassium voltage-gated channel subfamily H member 1	
LRRC7	Leucine-rich repeat-containing protein 7		*GABRB1*	Gamma-aminobutyric acid receptor subunit beta-1	GABA receptor(PC00023);acetylcholine receptor(PC00037)
SYNDIG1	Synapse differentiation-inducing gene protein 1		*GRIK2*	Glutamate receptor ionotropic, kainate 5	

NEURL1	E3 ubiquitin-protein ligase NEURL1	ubiquitin-protein ligase(PC00234)	*SLC8A1*	Sodium/calcium exchanger 1	
ANKS1B	Ankyrin repeat and sterile alpha motif domain-containing protein 1B	transmembrane receptor regulatory/adaptor protein(PC00226)	*ATAD1*	ATPase family AAA domain-containing protein 1	
SLC6A6	Sodium- and chloride-dependent taurine transporter	cation transporter(PC00068)	*SORCS3*	VPS10 domain-containing receptor SorCS3	receptor(PC00197);transporter(PC00227)
KCNB1	Potassium voltage-gated channel subfamily B member 1		*GABRG2*	Gamma-aminobutyric acid receptor subunit gamma-2	GABA receptor(PC00023);acetylcholine receptor(PC00037)
DGKI	Diacylglycerol kinase iota	kinase(PC00137)	*GRID1*	Glutamate receptor ionotropic, delta-1	
IGSF21	Immunoglobulin superfamily member 21		*HOMER1*	Homer protein homolog 1	
DISC1	Disrupted in schizophrenia 1 protein		*BAALC*	Brain and acute leukemia cytoplasmic protein	
CNTN1	Contactin-1		*ANK1*	Ankyrin-1	
GABRR2	Gamma-aminobutyric acid receptor subunit rho-2	GABA receptor(PC00023);acetylcholine receptor(PC00037)	*FARP1*	FERM, ARHGEF and pleckstrin domain-containing protein 1	
LIN7A	Protein lin-7 homolog A	cell adhesion molecule(PC00069);cell junction protein(PC00070)	*GRIA1*	Glutamate receptor 1	
GRIN2B	Glutamate receptor ionotropic, NMDA 2B		*CPEB4*	Cytoplasmic polyadenylation element-binding protein 4	mRNA polyadenylation factor(PC00146)
ACTN2	Alpha-actinin-2		*GABRB2*	Gamma-aminobutyric acid receptor subunit beta-2	GABA receptor(PC00023);acetylcholine receptor(PC00037)
DRD3	D(3) dopamine receptor	G-protein coupled receptor(PC00021)	*PSD3*	PH and SEC7 domain-containing protein 3	
TENM2	Teneurin-2		*DNM3*	Dynamin-3	hydrolase(PC00121);microtubule family cytoskeletal protein(PC00157);small GTPase(PC00208)
NRP1	Neuropilin-1		*GABRG3*	Gamma-aminobutyric acid receptor subunit gamma-3	GABA receptor(PC00023);acetylcholine receptor(PC00037)
CDH10	Cadherin-10		*GABBR2*	Gamma-aminobutyric acid type B receptor subunit 2	
RGS7BP	Regulator of G-protein signaling 7-binding protein		*GRIK2*	Glutamate receptor ionotropic, kainate 2	

KCNC2	Potassium voltage-gated channel subfamily C member 2		*GRIN2A*	Glutamate receptor ionotropic, NMDA 2A	
EPHA4	Ephrin type-A receptor 4		*PTPRO*	Receptor-type tyrosine-protein phosphatase O	
NRCAM	Neuronal cell adhesion molecule		*CLSTN2*	Calsyntenin-2	calcium-binding protein(PC00060);cell adhesion molecule(PC00069)
GRM5	Metabotropic glutamate receptor 5	G-protein coupled receptor(PC00021)	*GRIK4*	Glutamate receptor ionotropic, kainate 4	
GSG1L	Germ cell-specific gene 1-like protein	cytoskeletal protein(PC00085)	*ARHGAP32*	Rho GTPase-activating protein 32	
GRID2	Glutamate receptor ionotropic, delta-2		*GRIP1*	Glutamate receptor-interacting protein 1	
ADRA1A	Alpha-1A adrenergic receptor	G-protein coupled receptor(PC00021)	*LRRC4B*	Leucine-rich repeat-containing protein 4B	
LZTS1	Leucine zipper putative tumor suppressor 1		*DLG2*	Disks large homolog 2	transmembrane receptor regulatory/adaptor protein(PC00226)
KCNMA1	Calcium-activated potassium channel subunit alpha-1		*SHANK2*	SH3 and multiple ankyrin repeat domains protein 2	
SRGAP2	SLIT-ROBO Rho GTPase-activating protein 2	G-protein modulator(PC00022)	*SYNE1*	Nesprin-1	
DLGAP1	Disks large-associated protein 1	transmembrane receptor regulatory/adaptor protein(PC00226)	*CACNG4*	Voltage-dependent calcium channel gamma-4 subunit	voltage-gated calcium channel(PC00240)
SHISA9	Protein shisa-9		*GRM7*	Metabotropic glutamate receptor 7	G-protein coupled receptor(PC00021)
ANK2	Ankyrin-2		*TMEM108*	Transmembrane protein 108	
NRG1	Pro-neuregulin-1, membrane-bound isoform	growth factor(PC00112)	*DLGAP2*	Disks large-associated protein 2	transmembrane receptor regulatory/adaptor protein(PC00226)
CACNA1C	Voltage-dependent L-type calcium channel subunit alpha-1C		*NRP2*	Neuropilin-2;NRP2;ortholog	
OPRM1	Mu-type opioid receptor	G-protein coupled receptor(PC00021)	*TIAM1*	T-lymphoma invasion and metastasis-inducing protein 1	
LRFN5	Leucine-rich repeat and fibronectin type-III domain-containing protein 5		*GPHN*	Gephyrin;GPHN;ortholog	
OPRD1	Delta-type opioid receptor	G-protein coupled receptor(PC00021)	*ATP2B2*	Plasma membrane calcium-transporting ATPase 2	cation transporter(PC00068);hydrolase(PC00121);ion

					channel(PC00133)
ERBB4	Receptor tyrosine-protein kinase erbB-4				

Table S5

Table S5: Results from the PANTHER statistical overrepresentation test using the logistic regression classifier for the feature importance metric on the Dalhousie University (Halifax) sample. The selected sample of variant associated genes were those for which the absolute value of their regression coefficient was in the top quartile.						
	N Ref	N Obs	N Exp	Factor	p (Raw)	FDR
GO cellular component complete						
postsynaptic membrane (GO:0045211)	190	88	48.69	1.81	7.10E-06	3.01E-03
synaptic membrane (GO:0097060)	252	110	64.57	1.7	5.28E-06	2.98E-03
axon (GO:0030424)	303	122	77.64	1.57	3.37E-05	9.52E-03
postsynapse (GO:0098794)	327	125	83.79	1.49	1.56E-04	2.40E-02
synapse (GO:0045202)	618	230	158.36	1.45	8.23E-07	1.39E-03
synapse part (GO:0044456)	494	177	126.58	1.4	1.02E-04	1.93E-02
neuron projection (GO:0043005)	644	227	165.02	1.38	2.18E-05	7.39E-03
cell junction (GO:0030054)	634	218	162.46	1.34	1.22E-04	2.08E-02
neuron part (GO:0097458)	871	299	223.18	1.34	4.61E-06	3.91E-03
plasma membrane part (GO:0044459)	1408	432	360.78	1.2	2.99E-04	4.23E-02
plasma membrane (GO:0005886)	2407	712	616.77	1.15	4.44E-05	9.41E-03
cell periphery (GO:0071944)	2461	727	630.6	1.15	3.87E-05	9.37E-03
GO biological process complete						
neuron development (GO:0048666)	406	154	104.03	1.48	3.01E-05	4.36E-02
neuron differentiation (GO:0030182)	481	180	123.25	1.46	1.25E-05	2.72E-02
neurogenesis (GO:0022008)	777	274	199.1	1.38	2.35E-06	1.53E-02
generation of neurons (GO:0048699)	730	255	187.05	1.36	1.10E-05	3.57E-02
cell development (GO:0048468)	788	273	201.92	1.35	8.25E-06	3.59E-02
nervous system development (GO:0007399)	1103	376	282.63	1.33	3.27E-07	4.27E-03
cell differentiation (GO:0030154)	1589	494	407.16	1.21	2.71E-05	4.42E-02

cellular developmental process (GO:0048869)	1634	507	418.69	1.21	2.47E-05	4.61E-02
system development (GO:0048731)	1969	601	504.53	1.19	1.24E-05	3.24E-02

Table S6

Table S6: This table displays the top result of the statistical overrepresentation test for the gene ontology cellular component analysis on the Dalhousie sample. The postsynaptic membrane was the function annotation for which we found the highest enrichment factor in comparison to the reference set.					
Gene ID	Gene	PANTHER Protein Class	Gene ID	Gene	PANTHER Protein Class
LRFN2	Leucine-rich repeat and fibronectin type-III domain-containing protein 2		*OPRD1*	Delta-type opioid receptor	G-protein coupled receptor(PC00021)
CTNNA2	Catenin alpha-2	cell adhesion molecule(PC00069)<br><br>non-motor actin binding protein(PC00165)	*ERBB4*	Receptor tyrosine-protein kinase erbB-4	
CADPS<br><br>*CAPS2*	Calcium-dependent secretion activator 2	calcium-binding protein(PC00060)	*ANK3*	Ankyrin-3	
GRIP2	Glutamate receptor-interacting protein 2		*ADCY8*	Adenylate cyclase type 8	
IQSEC3	IQ motif and SEC7 domain-containing protein 3		*CLSTN1*	Calsyntenin-1	calcium-binding protein(PC00060)<br><br>cell adhesion molecule(PC00069)
CDH9	Cadherin-9		*GABRR1*	Gamma-aminobutyric acid receptor subunit rho-1	GABA receptor(PC00023)<br><br>acetylcholine receptor(PC00037)
CHRM3	Muscarinic acetylcholine receptor M3	G-protein coupled receptor(PC00021)	*LRRC4C*	Leucine-rich repeat-containing protein 4C	
ADAM22	Disintegrin and metalloproteinase domain-containing protein 22	metalloprotease(PC00153)	*KCNC1*	Potassium voltage-gated channel subfamily C member 1	
LRRC7	Leucine-rich repeat-containing protein 7		*KCNH1*	Potassium voltage-gated channel subfamily H member 1	
EPHA7	Ephrin type-A receptor 7		*GABRB1*	Gamma-aminobutyric acid receptor subunit beta-1	GABA receptor(PC00023)<br><br>acetylcholine receptor(PC00037)
ANKS1B	Ankyrin repeat and sterile alpha motif domain-containing protein 1B	transmembrane receptor regulatory/adaptor protein(PC00226)	*GRIK2*	Glutamate receptor ionotropic, kainate 5	
SLC6A6	Sodium- and chloride-dependent taurine transporter	cation transporter(PC00068)	*SLC8A1*	Sodium/calcium exchanger 1	

DGKI	Diacylglycerol kinase iota;DGKI;ortholog	kinase(PC00137)	*CPEB3*	Cytoplasmic polyadenylation element-binding protein 3	mRNA polyadenylation factor(PC00146)
LRRTM3	Leucine-rich repeat transmembrane neuronal protein 3	extracellular matrix protein(PC00102)<br><br>receptor(PC00197)	*COL13A1*	Collagen alpha-1(XIII) chain	
IGSF21	Immunoglobulin superfamily member 21		*ATAD1*	ATPase family AAA domain-containing protein 1	
DISC1	Disrupted in schizophrenia 1 protein		*SLC6A1*	Sodium- and chloride-dependent GABA transporter 1	cation transporter(PC00068)
CNTN1	Contactin-1		*GRID1*	Glutamate receptor ionotropic, delta-1	
GABRR2	Gamma-aminobutyric acid receptor subunit rho-2	GABA receptor(PC00023)<br><br>acetylcholine receptor(PC00037)	*HOMER1*	Homer protein homolog 1	
LIN7A	Protein lin-7 homolog A	cell adhesion molecule(PC00069)<br><br>cell junction protein(PC00070)	*NLGN1*	Neuroligin-1	
GRIN2B	Glutamate receptor ionotropic, NMDA 2B		*ANK1*	Ankyrin-1	
LHFPL4	LHFPL tetraspan subfamily member 4 protein		*P2RY1*	P2Y purinoceptor 1	
GRIK3	Glutamate receptor ionotropic, kainate 3		*GLRB*	Glycine receptor subunit beta	GABA receptor(PC00023)<br><br>acetylcholine receptor(PC00037)
TENM2	Teneurin-2		*GRIA1*	Glutamate receptor 1	
NRP1	Neuropilin-1		*DRD2*	D(2) dopamine receptor	G-protein coupled receptor(PC00021)
CDH10	Cadherin-10		*GABRB2*	Gamma-aminobutyric acid receptor subunit beta-2	GABA receptor(PC00023)<br><br>acetylcholine receptor(PC00037)
PICALM	Phosphatidylinositol-binding clathrin assembly protein	vesicle coat protein(PC00235)	*PSD3*	PH and SEC7 domain-containing protein 3	
KCNC2	Potassium voltage-gated channel subfamily C member 2		*DNM3*	Dynamin-3	hydrolase(PC00121)<br><br>microtubule family cytoskeletal protein(PC00157)<br><br>small GTPase(PC00208)

GRIN3A	Glutamate receptor ionotropic, NMDA 3A		*GABBR2*	Gamma-aminobutyric acid type B receptor subunit 2	
EPHA4	Ephrin type-A receptor 4		*GRIK2*	Glutamate receptor ionotropic, kainate 2	
SLC6A3	Sodium-dependent dopamine transporter	cation transporter(PC00068)	*PTPRO*	Receptor-type tyrosine-protein phosphatase O	
NRCAM	Neuronal cell adhesion molecule		*CLSTN2*	Calsyntenin-2	calcium-binding protein(PC00060)<br><br>cell adhesion molecule(PC00069)
GRID2	Glutamate receptor ionotropic, delta-2		*GRIK4*	Glutamate receptor ionotropic, kainate 4	
LZTS1	Leucine zipper putative tumor suppressor 1		*ARRB1*	Beta-arrestin-1	enzyme modulator(PC00095)
KCNMA1	Calcium-activated potassium channel subunit alpha-1		*SCRIB*	Protein scribble homolog	
ADORA1	Adenosine receptor A1	G-protein coupled receptor(PC00021)	*GRIP1*	Glutamate receptor-interacting protein 1	
EPHB2	Ephrin type-B receptor 2		*CHRNA1*	Acetylcholine receptor subunit alpha	GABA receptor(PC00023)<br><br>acetylcholine receptor(PC00037)
KCND3	Potassium voltage-gated channel subfamily D member 3		*DLG2*	Disks large homolog 2	transmembrane receptor regulatory/adaptor protein(PC00226)
ANK2	Ankyrin-2		*SHANK2*	SH3 and multiple ankyrin repeat domains protein 2	
NRG1	Pro-neuregulin-1, membrane-bound isoform	growth factor(PC00112)	*SYNE1*	Nesprin-1	
SSPN	Sarcospan		*GRM7*	Metabotropic glutamate receptor 7	G-protein coupled receptor(PC00021)
CACNA1C	Voltage-dependent L-type calcium channel subunit alpha-1C		*TMEM108*	Transmembrane protein 108	
MUSK	Muscle, skeletal receptor tyrosine-protein kinase		*DLGAP2*	Disks large-associated protein 2	transmembrane receptor regulatory/adaptor protein(PC00226)
OPRM1	Mu-type opioid receptor	G-protein coupled receptor(PC00021)	*NRP2*	Neuropilin-2	
FAIM2	Protein lifeguard 2		*ATP2B2*	Plasma membrane calcium-transporting	cation

				ATPase 2	transporter(PC00068)<br>hydrolase(PC00121)<br>ion channel(PC00133)

Table S7

Table S7: This table displays the top result of the statistical overrepresentation test for the gene ontology biological process analysis on the Dalhousie sample. Neuron development was the function annotation for which we found the highest enrichment factor in comparison to the reference set.					
Gene ID	Gene	PANTHER Protein Class	Gene ID	Gene	
CDH23	Dihydropyrimidinase-related protein 2	metalloprotease(PC00153)	*EFNA5*	Ephrin-A5	
ABL1	Tyrosine-protein kinase ABL1		*NTNG1*	Netrin-G1	
CTNNA2	Catenin alpha-2	cell adhesion molecule(PC00069)<br><br>non-motor actin binding protein(PC00165)	*ROBO3*	Roundabout homolog 3	
PREX2	Phosphatidylinositol 3,4,5-trisphosphate-dependent Rac exchanger 2 protein	guanyl-nucleotide exchange factor(PC00113)	*VLDLR*		
TNFRSF21	Tumor necrosis factor receptor superfamily member 21		*MTCH1*	Mitochondrial carrier homolog 1	
PLXNC1	Plexin-C1		*SECISBP2*		
GLI2	Zinc finger protein GLI2		*ITGA1*	Integrin alpha-1	
NEUROD1	Neurogenic differentiation factor 1	basic helix-loop-helix transcription factor(PC00055)<br><br>nuclease(PC00170)	*CYFIP2*	Cytoplasmic FMR1-interacting protein 2	
LAMA2	Laminin subunit alpha-2	extracellular matrix linker protein(PC00101)<br><br>protease inhibitor(PC00191)<br><br>receptor(PC00197)	*CNR1*	Cannabinoid receptor 1	
DPYSL3	Dihydropyrimidinase-related protein 3	metalloprotease(PC00153)	*EPHB1*		

GPM6A	Neuronal membrane glycoprotein M6-a	myelin protein(PC00161)	*GRB10*	Growth factor receptor-bound protein 10	
SEMA5B	Semaphorin-5B		*SLC1A3*	Excitatory amino acid transporter 1	
RELN	Reelin		*NTF3*	Neurotrophin-3	
JAM3	Junctional adhesion molecule C		*GABRB1*	Gamma-aminobutyric acid receptor subunit beta-1	
JADE2	E3 ubiquitin-protein ligase Jade-2	nucleic acid binding(PC00171)<br><br>zinc finger transcription factor(PC00244)	*JAK2*		
PTK2	Focal adhesion kinase 1		*COBL*	Protein cordon-bleu	
NTM	Neurotrimin		*CHL1*		
UGT8	2-hydroxyacylsphingosine 1-beta-galactosyltransferase		*PHGDH*	D-3-phosphoglycerate dehydrogenase	
EPHA7	Ephrin type-A receptor 7		*CNTN6*	Contactin-6	
THRB	Thyroid hormone receptor beta	C4 zinc finger nuclear receptor(PC00169)<br><br>nucleic acid binding(PC00171)<br><br>receptor(PC00197)	*PAK2*		
LRP2	Low-density lipoprotein receptor-related protein 2		*COL25A1*		
NREP	Neuronal regeneration-related protein		*NLGN1*	Neuroligin-1	
BOC	Brother of CDO		*IL6*	Interleukin-6	
PCDH15	Protocadherin-15		*FIG4*		

ALCAM	CD166 antigen	immunoglobulin superfamily cell adhesion molecule(PC00125)<br><br>receptor(PC00197)	*DCDC2*		
NTN4	Netrin-4	extracellular matrix linker protein(PC00101)<br><br>protease inhibitor(PC00191)<br><br>receptor(PC00197)	*DRD2*	D(2) dopamine receptor	
CPN1	Copine-1		*ITGB1*	Integrin beta-1	
LGI1	Leucine-rich glioma-inactivated protein 1		*OPCML*		
CNTN1	Contactin-1		*GABRB2*	Gamma-aminobutyric acid receptor subunit beta-2	
DGKG	Diacylglycerol kinase gamma	kinase(PC00137)	*MYPN*	Myopalladin	
GDPD5	Glycerophosphodiester phosphodiesterase domain-containing protein 5		*NOV*	Plexin-A1	
IGSF9	Protein turtle homolog A	transmembrane receptor regulatory/adaptor protein(PC00226)	*SEMA5A*	Semaphorin-5A	
FGFR1	Fibroblast growth factor receptor 1		*OLFM3*	Noelin-3	
UHMK1	Serine/threonine-protein kinase Kist		*PLXNA4*	Plexin-A4	
TENM2	Teneurin-2		*CDNF*		
NRP1	Neuropilin-1		*NYAP2*		
STK3	Serine/threonine-protein kinase 24		*MATN2*	Matrilin-2	receptor(PC00197)

CNTN4	Contactin-4	transmembrane receptor regulatory/adaptor protein(PC00226)	*CDHR1*		
PICALM	Phosphatidylinositol-binding clathrin assembly protein	vesicle coat protein(PC00235)	*PTPRO*		
AUTS2	Autism susceptibility gene 2 protein		*FEZ2*		
SGK1	Serine/threonine-protein kinase Sgk1	annexin(PC00050)<br>calmodulin(PC00061)<br>non-receptor serine/threonine protein kinase(PC00167)<br>transfer/carrier protein(PC00219)	*TRAK1*	Trafficking kinesin-binding protein 1	
GRIN3A	Glutamate receptor ionotropic, NMDA 3A		*PALLD*	Palladin	
RTN4	Reticulon-4		*TENM3*	Teneurin-3	
CNTNAP2	Contactin-associated protein-like 2		*TNN*	Tenascin-N	
EPHA4	Ephrin type-A receptor 4		*SCRIB*		
NRCAM	Neuronal cell adhesion molecule		*FSHR*	Follicle-stimulating hormone receptor	
DPYSL4	Dihydropyrimidinase-related protein 4	metalloprotease(PC00153)	*EPHA6*		
PRKG1	cGMP-dependent protein kinase 1		*TNIK*		
PAX2	Paired box protein Pax-2		*GRIP1*		
ROBO2	Roundabout homolog 2		*PRKCQ*	Protein kinase C theta type	

SLIT3	Slit homolog 3 protein		*UNC5C*	Netrin receptor UNC5C	receptor(PC00197)
NCAM1	Neural cell adhesion molecule 1		*ALK*		
PTPN11	Tyrosine-protein phosphatase non-receptor type 11		*LMX1A*	LIM homeobox transcription factor 1-alpha	
NFIB	Nuclear factor 1 B-type	nucleic acid binding(PC00171)<br><br>transcription factor(PC00218)	*GFRA1*		
ROBO1	Roundabout homolog 1		*SHANK2*		
NFASC	Neurofascin		*UBE4B*	Ubiquitin conjugation factor E4 B	
CDH23	Cadherin-23		*TNR*	Tenascin-R	
CRMP1	Dihydropyrimidinase-related protein 1	metalloprotease(PC00153)	*MAP1B*	Microtubule-associated protein 1B	
FARP2	FERM, ARHGEF and pleckstrin domain-containing protein 2		*RAP1GAP*	Rap1 GTPase-activating protein 1	
RET	Proto-oncogene tyrosine-protein kinase receptor Ret		*SLIT2*	Slit homolog 2 protein	
EPHB2	Ephrin type-B receptor 2		*APBB2*		
GLI3	Transcriptional activator GLI3		*NRXN1*	Neurexin-1	
PTPRK	Receptor-type tyrosine-protein phosphatase kappa	protein phosphatase(PC00195)<br><br>receptor(PC00197)	*SEMA6A*	Semaphorin-6A	

ELAVL4	ELAV-like protein 4		*PARD3*		
VANGL2	Vang-like protein 2		*SEMA3C*	Semaphorin-3C	
MYT1L	Myelin transcription factor 1-like protein	zinc finger transcription factor(PC00244)	*ADGRB3*	Adhesion G protein-coupled receptor B3	
SH3GL2	Endophilin-A1		*TMEM108*		
TGFB2	Transforming growth factor beta-2	growth factor(PC00112)	*KIRREL3*		
BTG2	Protein BTG2		*NRP2*	Neuropilin-2	
CXCL12	Stromal cell-derived factor 1		*NCK2*		
DSCAML1	Down syndrome cell adhesion molecule-like protein 1		*DCLK2*		
TENM4	Teneurin-4		*PPT1*	Palmitoyl-protein thioesterase 1	esterase(PC00097)
GAB2	GRB2-associated-binding protein 2	transmembrane receptor regulatory/adaptor protein(PC00226)	*ITGA4*	Integrin alpha-4	
EXT1	Exostosin-1	glycosyltransferase(PC00111)	*MYO7A*	Unconventional myosin-VIIa	
DAB1	Disabled homolog 1	signaling molecule(PC00207)	*PTK2B*		
DOCK10	Dedicator of cytokinesis protein 10	guanyl-nucleotide exchange factor(PC00113)	*EGFR*		
ANK3	Ankyrin-3		*CTNND2*	Catenin delta-2	

Table S8

Table S8: Results from the PANTHER gene ontology analysis performed on genes selected from the logistic regression analysis on the Würzburg sample.						
Functional Class	N Ref	N Obs	N Exp	Factor	p (Raw)	FDR
synaptic membrane (GO:0097060)	252	105	65.55	1.6	5.94E-05	2.52E-02
synapse (GO:0045202)	618	222	160.76	1.38	2.57E-05	1.45E-02
plasma membrane bounded cell projection (GO:0120025)	1016	346	264.29	1.31	4.17E-06	7.06E-03
cell projection (GO:0042995)	1055	351	274.44	1.28	2.08E-05	1.76E-02

Table S9

Table S9: This table displays the top result of the statistical overrepresentation test for the gene ontology cellular component analysis on the Würzburg sample. Synaptic membrane was the function annotation for which we found the highest enrichment factor in comparison to the reference set.

LRFN2	Leucine-rich repeat and fibronectin type-III domain-containing protein 2		*GABRR1*	Gamma-aminobutyric acid receptor subunit rho-1	GABA receptor(PC00023);acetylcholine receptor(PC00037)
CNTN2	Contactin-2	transmembrane receptor regulatory/adaptor protein(PC00226)	*NTNG1*	Netrin-G1	extracellular matrix linker protein(PC00101);protease inhibitor(PC00191);receptor(PC00197)
CTNNA2	Catenin alpha-2	cell adhesion molecule(PC00069);non-motor actin binding protein(PC00165)	*LRRC4C*	Leucine-rich repeat-containing protein 4C	
NTNG2	Netrin-G2	extracellular matrix linker protein(PC00101);protease inhibitor(PC00191);receptor(PC00197)	*SLC6A11*	Sodium- and chloride-dependent GABA transporter 3;	cation transporter(PC00068)
GPM6A	Neuronal membrane glycoprotein M6-a	myelin protein(PC00161)	*ADGRB1*	Adhesion G protein-coupled receptor B1	G-protein coupled receptor(PC00021);antibacterial response protein(PC00051);protease(PC00190)
CDH9	Cadherin-9		*ADAM23*	Disintegrin and metalloproteinase domain-containing protein 23	metalloprotease(PC00153)
CHRM3	Muscarinic acetylcholine receptor M3	G-protein coupled receptor(PC00021)	*RIMS2*	Regulating synaptic membrane exocytosis protein 2	G-protein modulator(PC00022)
GRM6	Metabotropic glutamate receptor 6	G-protein coupled receptor(PC00021)	*KCNH1*	Potassium voltage-gated channel subfamily H member 1	

LRRC7	Leucine-rich repeat-containing protein 7		*GRIK2*	Glutamate receptor ionotropic, kainate 5	
NEURL1	E3 ubiquitin-protein ligase NEURL1	ubiquitin-protein ligase(PC00234)	*SLC8A1*	Sodium/calcium exchanger 1	
ANKS1B	Ankyrin repeat and sterile alpha motif domain-containing protein 1B	transmembrane receptor regulatory/adaptor protein(PC00226)	*COL13A1*	Collagen alpha-1(XIII) chain	
DNM1L	Dynamin-1-like protein	hydrolase(PC00121);microtubule family cytoskeletal protein(PC00157);small GTPase(PC00208)	*ATP2B2*	Plasma membrane calcium-transporting ATPase 4	cation transporter(PC00068);hydrolase(PC00121);ion channel(PC00133)
SLC6A6	Sodium- and chloride-dependent taurine transporter	cation transporter(PC00068)	*CNTN6*	Contactin-6	transmembrane receptor regulatory/adaptor protein(PC00226)
IGSF21	Immunoglobulin superfamily member 21		*SLC6A1*	Sodium- and chloride-dependent GABA transporter 1	cation transporter(PC00068)
DISC1	Disrupted in schizophrenia 1 protein		*GRID1*	Glutamate receptor ionotropic, delta-1	
CNTN1	Contactin-1		*FAM196A*	Protein FAM196A	
GABRR2	Gamma-aminobutyric acid receptor subunit rho-2	GABA receptor(PC00023);acetylcholine receptor(PC00037)	*NLGN1*	Neuroligin-1	
LIN7A	Protein lin-7 homolog A	cell adhesion molecule(PC00069);cell junction protein(PC00070)	*ANK1*	Ankyrin-1	
GRIN2B	Glutamate receptor ionotropic, NMDA 2B		*GLRB*	Glycine receptor subunit beta	GABA receptor(PC00023);acetylcholine receptor(PC00037)
LHFPL4	LHFPL tetraspan subfamily member 4 protein		*GRIA1*	Glutamate receptor 1	

SYT6	Synaptotagmin-6	membrane trafficking regulatory protein(PC00151)	*DENND1A*	DENN domain-containing protein 1A	
ACTN2	Alpha-actinin-2		*CPEB4*	Cytoplasmic polyadenylation element-binding protein 4	mRNA polyadenylation factor(PC00146)
TENM2	Teneurin-2		*GABRB2*	Gamma-aminobutyric acid receptor subunit beta-2	GABA receptor(PC00023);acetylcholine receptor(PC00037)
HIP1	Huntingtin-interacting protein 1	non-motor actin binding protein(PC00165)	*PSD3*	PH and SEC7 domain-containing protein 3	
NRP1	Neuropilin-1		*DNM3*	Dynamin-3	hydrolase(PC00121);microtubule family cytoskeletal protein(PC00157);small GTPase(PC00208)
NCSTN	Nicastrin		*GABBR2*	Gamma-aminobutyric acid type B receptor subunit 2	
GRIN3A	Glutamate receptor ionotropic, NMDA 3A		*ERC2*	ERC protein 2	G-protein modulator(PC00022);membrane traffic protein(PC00150)
EPHA4	Ephrin type-A receptor 4		*GRIK2*	Glutamate receptor ionotropic, kainate 2	
SLC6A3	Sodium-dependent dopamine transporter	cation transporter(PC00068)	*SYT1*	Synaptotagmin-1	membrane trafficking regulatory protein(PC00151)
NRCAM	Neuronal cell adhesion molecule		*CLSTN3*	Calsyntenin-3	calcium-binding protein(PC00060);cell adhesion molecule(PC00069)
GRM5	Metabotropic glutamate receptor 5	G-protein coupled receptor(PC00021)	*CLSTN2*	Calsyntenin-2	calcium-binding protein(PC00060);cell adhesion molecule(PC00069)
CPE	Carboxypeptidase E		*GRIK4*	Glutamate receptor ionotropic, kainate 4	

GRID2	Glutamate receptor ionotropic, delta-2		*CNIH3*	Protein cornichon homolog 3	membrane traffic protein(PC00150);membrane-bound signaling molecule(PC00152)
LZTS1	Leucine zipper putative tumor suppressor 1		*ZDHHC17*	Palmitoyltransferase ZDHHC17	
ADCY1	Adenylate cyclase type 1		*ARHGAP32*	Rho GTPase-activating protein 32	
KCNMA1	Calcium-activated potassium channel subunit alpha-1		*CNTN5*	Contactin-5	
ERC1	ELKS/Rab6-interacting/CAST family member 1	G-protein modulator(PC00022);membrane traffic protein(PC00150)	*GRIP1*	Glutamate receptor-interacting protein 1	
KCNJ3	G protein-activated inward rectifier potassium channel 1		*DLG2*	Disks large homolog 2	transmembrane receptor regulatory/adaptor protein(PC00226)
DTNBP1	Dysbindin;DTNBP1		*PDLIM5*	PDZ and LIM domain protein 5	non-motor actin binding protein(PC00165)
TANC1	Protein TANC1		*SHANK2*	SH3 and multiple ankyrin repeat domains protein 2	
APBA1	Amyloid-beta A4 precursor protein-binding family A member 1	membrane trafficking regulatory protein(PC00151)	*CACNA1D*	Voltage-dependent L-type calcium channel subunit alpha-1D	
EPHB2	Ephrin type-B receptor 2		*SYNE1*	Nesprin-1	
CABP1	Calcium-binding protein 1		*GRIA4*	Glutamate receptor 4	
GRM1	Metabotropic glutamate receptor 1	G-protein coupled receptor(PC00021)	*NRXN1*	Neurexin-1	
KCND3	Potassium voltage-gated channel subfamily D member 3		*GRM7*	Metabotropic glutamate receptor 7	G-protein coupled receptor(PC00021)
ANK2	Ankyrin-2		*STXBP5*	Syntaxin-binding protein 5	membrane trafficking regulatory

					protein(PC00151)
NRG1	Pro-neuregulin-1, membrane-bound isoform	growth factor(PC00112)	*TMEM108*	Transmembrane protein 108	
CACNA1C	Voltage-dependent L-type calcium channel subunit alpha-1C		*DLGAP2*	Disks large-associated protein 2	transmembrane receptor regulatory/adaptor protein(PC00226)
MUSK	Muscle, skeletal receptor tyrosine-protein kinase		*NRP2*	Neuropilin-2;NRP2;ortholog	
OPRM1	Mu-type opioid receptor	G-protein coupled receptor(PC00021)	*CHRM2*	Muscarinic acetylcholine receptor M2	G-protein coupled receptor(PC00021)
ERBB4	Receptor tyrosine-protein kinase erbB-4		*UTRN*	Utrophin	
ANK3	Ankyrin-3		*ATP2B2*	Plasma membrane calcium-transporting ATPase 2	cation transporter(PC00068);hydrolase(PC00121);ion channel(PC00133)
ADCY8	Adenylate cyclase type 8				

Classification Analysis with Progressively Narrowed Lithium Response Definitions

The methods of this experiment are reported in the main body of the text. Results of the aggregate and site-level classification analyses across the four Alda score thresholding conditions are reported in Table S9.

Table S10

Table S10: Results for the aggregate and site level classification analyses using four different Alda score thresholds to separate responders and non-responders. Columns represent the different thresholds and values represent the mean value of the given statistic across five folds with a 95% empirical confidence interval. Asterisks in the rows that represent the Kappa statistic signify a p-value less than 0.01 in comparison to the simulated null classifier. *Abbreviations*: all sites (ALL; i.e. aggregate analysis), area under the receiver operating characteristic curve (AUC), positive predictive value (PPV), negative predictive value (NPV), F-1 score (F1).

Site	Statistic	Alda (0-5; 7-10)	Alda (0-5; 8-10)	Alda (0-4; 8-10)	Alda (0-3; 9-10)
Aggregate Analysis, LR Classifier					
ALL	AUC	0.57 (0.56, 0.59)	0.61 (0.59, 0.63)	0.62 (0.59, 0.65)	0.65 (0.59, 0.7)
ALL	Sensitivity	0.06 (0.05, 0.08)	0.01 (0.01, 0.02)	0.02 (0.01, 0.04)	0 (0, 0)
ALL	Specificity	0.97 (0.96, 0.98)	1 (1, 1)	1 (1, 1)	1 (1, 1)
ALL	PPV	0.5 (0.41, 0.58)	0.8 (0.41, 1.19)	1 (1, 1)	0 (0, 0)
ALL	NPV	0.7 (0.69, 0.7)	0.77 (0.77, 0.77)	0.74 (0.74, 0.75)	0.82 (0.82, 0.82)
ALL	F1	0.11 (0.09, 0.13)	0.03 (0.01, 0.05)	0.05 (0.02, 0.07)	0 (0, 0)
ALL	Kappa	0.04 (0.03, 0.06)	0.02 (0.01, 0.04)	0.03 (0.02, 0.05)	0 (0, 0)
Site Level Analysis, LR Classifier					
Barcelona	AUC	0.42 (0.25, 0.59)	0.52 (0.33, 0.72)	0.5 (0.41, 0.6)	0.53 (0.38, 0.68)
Barcelona	Sensitivity	0 (0, 0)	0 (0, 0)	0 (0, 0)	0 (0, 0)
Barcelona	Specificity	1 (1, 1)	1 (1, 1)	1 (1, 1)	1 (1, 1)
Barcelona	PPV	0 (0, 0)	0 (0, 0)	0 (0, 0)	0 (0, 0)
Barcelona	NPV	0.67 (0.66, 0.68)	0.72 (0.71, 0.73)	0.67 (0.65, 0.7)	0.74 (0.73, 0.76)
Barcelona	F1	0 (0, 0)	0 (0, 0)	0 (0, 0)	0 (0, 0)
Barcelona	Kappa	0 (0, 0)	0 (0, 0)	0 (0, 0)	0 (0, 0)

Cagliari	AUC	0.49 (0.41, 0.57)	0.64 (0.49, 0.79)	0.55 (0.45, 0.65)	0.49 (0.31, 0.67)
Cagliari	Sensitivity	0 (0, 0)	0 (0, 0)	0 (0, 0)	0 (0, 0)
Cagliari	Specificity	1 (1, 1)	1 (1, 1)	1 (1, 1)	1 (1, 1)
Cagliari	PPV	0 (0, 0)	0 (0, 0)	0 (0, 0)	0 (0, 0)
Cagliari	NPV	0.68 (0.67, 0.68)	0.83 (0.82, 0.84)	0.8 (0.79, 0.81)	0.9 (0.89, 0.9)
Cagliari	F1	0 (0, 0)	0 (0, 0)	0 (0, 0)	0 (0, 0)
Cagliari	Kappa	0 (0, 0)	0 (0, 0)	0 (0, 0)	0 (0, 0)
Geneva	AUC	0.46 (0.33, 0.59)	0.38 (0.09, 0.67)	0.38 (0.09, 0.67)	0.61 (0.39, 0.83)
Geneva	Sensitivity	0 (0, 0)	0 (0, 0)	0 (0, 0)	0 (0, 0)
Geneva	Specificity	1 (1, 1)	1 (1, 1)	1 (1, 1)	1 (1, 1)
Geneva	PPV	0 (0, 0)	0 (0, 0)	0 (0, 0)	0 (0, 0)
Geneva	NPV	0.75 (0.72, 0.78)	0.82 (0.79, 0.84)	0.79 (0.76, 0.82)	0.91 (0.85, 0.98)
Geneva	F1	0 (0, 0)	0 (0, 0)	0 (0, 0)	0 (0, 0)
Geneva	Kappa	0 (0, 0)	0 (0, 0)	0 (0, 0)	0 (0, 0)
Halifax	AUC	0.66 (0.62, 0.71)	0.71 (0.68, 0.73)	0.7 (0.65, 0.75)	0.67 (0.58, 0.76)
Halifax	Sensitivity	0.38 (0.29, 0.47)	0.15 (0.12, 0.17)	0.25 (0.21, 0.28)	0 (0, 0)
Halifax	Specificity	0.79 (0.75, 0.83)	0.97 (0.95, 0.99)	0.92 (0.85, 0.98)	1 (1, 1)
Halifax	PPV	0.62 (0.59, 0.64)	0.8 (0.69, 0.91)	0.76 (0.6, 0.91)	0 (0, 0)
Halifax	NPV	0.59 (0.56, 0.61)	0.61 (0.6, 0.62)	0.6 (0.59, 0.61)	0.67 (0.66, 0.67)
Halifax	F1	0.46 (0.39, 0.54)	0.25 (0.2, 0.29)	0.36 (0.33, 0.4)	0 (0, 0)
Halifax	Kappa	0.17 (0.12, 0.23)[a]	0.13 (0.09, 0.17)	0.18 (0.12, 0.23)[a]	0 (0, 0)
Japan	AUC	0.57 (0.41, 0.73)	0.55 (0.48, 0.63)	0.54 (0.48, 0.6)	0.59 (0.41, 0.77)
Japan	Sensitivity	0 (0, 0)	0 (0, 0)	0 (0, 0)	0 (0, 0)
Japan	Specificity	1 (1, 1)	1 (1, 1)	1 (1, 1)	1 (1, 1)
Japan	PPV	0 (0, 0)	0 (0, 0)	0 (0, 0)	0 (0, 0)
Japan	NPV	0.74 (0.73, 0.75)	0.78 (0.77, 0.78)	0.75 (0.75, 0.76)	0.75 (0.73, 0.77)
Japan	F1	0 (0, 0)	0 (0, 0)	0 (0, 0)	0 (0, 0)
Japan	Kappa	0 (0, 0)	0 (0, 0)	0 (0, 0)	0 (0, 0)
Mayo Clinic	AUC	0.39 (0.28, 0.5)	0.45 (0.32, 0.58)	0.45 (0.25, 0.65)	0.31 (0.09, 0.53)

Mayo Clinic	Sensitivity	0 (0, 0)	0 (0, 0)	0 (0, 0)	0 (0, 0)
Mayo Clinic	Specificity	1 (1, 1)	1 (1, 1)	1 (1, 1)	1 (1, 1)
Mayo Clinic	PPV	0 (0, 0)	0 (0, 0)	0 (0, 0)	0 (0, 0)
Mayo Clinic	NPV	0.74 (0.73, 0.75)	0.82 (0.8, 0.84)	0.78 (0.76, 0.81)	0.91 (0.87, 0.96)
Mayo Clinic	F1	0 (0, 0)	0 (0, 0)	0 (0, 0)	0 (0, 0)
Mayo Clinic	Kappa	0 (0, 0)	0 (0, 0)	0 (0, 0)	0 (0, 0)
Paris	AUC	0.39 (0.28, 0.5)	0.5 (0.33, 0.68)	0.48 (0.29, 0.68)	0.59 (0.3, 0.88)
Paris	Sensitivity	0 (0, 0)	0 (0, 0)	0 (0, 0)	0 (0, 0)
Paris	Specificity	1 (1, 1)	1 (1, 1)	1 (1, 1)	1 (1, 1)
Paris	PPV	0 (0, 0)	0 (0, 0)	0 (0, 0)	0 (0, 0)
Paris	NPV	0.8 (0.79, 0.8)	0.9 (0.89, 0.91)	0.88 (0.86, 0.89)	0.94 (0.92, 0.96)
Paris	F1	0 (0, 0)	0 (0, 0)	0 (0, 0)	0 (0, 0)
Paris	Kappa	0 (0, 0)	0 (0, 0)	0 (0, 0)	0 (0, 0)
Poznan	AUC	0.5 (0.36, 0.64)	0.45 (0.37, 0.54)	0.6 (0.35, 0.85)	0.61 (0.52, 0.71)
Poznan	Sensitivity	0.85 (0.8, 0.9)	0.03 (-0.03, 0.08)	1 (1, 1)	1 (1, 1)
Poznan	Specificity	0.19 (0.13, 0.24)	0.88 (0.74, 1.01)	0 (0, 0)	0 (0, 0)
Poznan	PPV	0.53 (0.5, 0.56)	0.1 (-0.1, 0.3)	0.6 (0.58, 0.61)	0.65 (0.62, 0.69)
Poznan	NPV	0.53 (0.37, 0.69)	0.51 (0.46, 0.56)	NA	NA
Poznan	F1	0.66 (0.62, 0.69)	0.04 (-0.04, 0.13)	0.75 (0.73, 0.76)	0.79 (0.76, 0.81)
Poznan	Kappa	0.04 (-0.07, 0.15)	-0.1 (-0.25, 0.06)	0 (0, 0)	0 (0, 0)
Romania	AUC	0.56 (0.51, 0.61)	0.44 (0.34, 0.54)	0.44 (0.32, 0.56)	0.52 (0.19, 0.84)
Romania	Sensitivity	0 (0, 0)	0 (0, 0)	0 (0, 0)	0 (0, 0)
Romania	Specificity	1 (1, 1)	1 (1, 1)	1 (1, 1)	1 (1, 1)
Romania	PPV	0 (0, 0)	0 (0, 0)	0 (0, 0)	0 (0, 0)
Romania	NPV	0.77 (0.76, 0.79)	0.9 (0.88, 0.92)	0.89 (0.87, 0.91)	0.96 (0.93, 0.98)
Romania	F1	0 (0, 0)	0 (0, 0)	0 (0, 0)	0 (0, 0)
Romania	Kappa	0 (0, 0)	0 (0, 0)	0 (0, 0)	0 (0, 0)
San Diego	AUC	0.51 (0.42, 0.59)	0.51 (0.36, 0.66)	0.5 (0.34, 0.67)	0.48 (0.32, 0.64)
San Diego	Sensitivity	0 (0, 0)	0 (0, 0)	0 (0, 0)	0 (0, 0)

San Diego	Specificity	1 (1, 1)	1 (1, 1)	1 (1, 1)	1 (1, 1)
San Diego	PPV	0 (0, 0)	0 (0, 0)	0 (0, 0)	0 (0, 0)
San Diego	NPV	0.88 (0.87, 0.89)	0.95 (0.95, 0.95)	0.94 (0.94, 0.94)	0.96 (0.96, 0.96)
San Diego	F1	0 (0, 0)	0 (0, 0)	0 (0, 0)	0 (0, 0)
San Diego	Kappa	0 (0, 0)	0 (0, 0)	0 (0, 0)	0 (0, 0)
Sweden	AUC	0.47 (0.43, 0.52)	0.47 (0.44, 0.5)	0.45 (0.4, 0.49)	0.42 (0.33, 0.5)
Sweden	Sensitivity	0.37 (0.3, 0.44)	0 (0, 0)	0 (0, 0)	0 (0, 0)
Sweden	Specificity	0.66 (0.56, 0.77)	1 (1, 1)	0.98 (0.96, 1)	1 (1, 1)
Sweden	PPV	0.52 (0.43, 0.61)	0 (0, 0)	0 (0, 0)	0 (0, 0)
Sweden	NPV	0.52 (0.48, 0.57)	0.64 (0.63, 0.64)	0.58 (0.57, 0.59)	0.68 (0.67, 0.69)
Sweden	F1	0.43 (0.36, 0.49)	0 (0, 0)	0 (0, 0)	0 (0, 0)
Sweden	Kappa	0.03 (-0.09, 0.15)	0 (0, 0)	-0.02 (-0.04, 0)	0 (0, 0)
Sydney	AUC	0.35 (0.18, 0.51)	0.16 (0.03, 0.28)	0.27 (0.15, 0.4)	0.49 (0.17, 0.8)
Sydney	Sensitivity	0 (0, 0)	0 (0, 0)	0 (0, 0)	0 (0, 0)
Sydney	Specificity	0.98 (0.93, 1.02)	0.98 (0.93, 1.02)	0.98 (0.93, 1.02)	1 (1, 1)
Sydney	PPV	0 (0, 0)	0 (0, 0)	0 (0, 0)	0 (0, 0)
Sydney	NPV	0.79 (0.76, 0.81)	0.81 (0.79, 0.84)	0.79 (0.77, 0.81)	0.87 (0.85, 0.9)
Sydney	F1	0 (0, 0)	0 (0, 0)	0 (0, 0)	0 (0, 0)
Sydney	Kappa	-0.03 (-0.08, 0.03)	-0.03 (-0.08, 0.03)	-0.03 (-0.09, 0.03)	0 (0, 0)
Taiwan	AUC	0.4 (0.23, 0.57)	0.35 (0.13, 0.58)	0.3 (0.14, 0.46)	0.07 (-0.02, 0.15)
Taiwan	Sensitivity	0 (0, 0)	0 (0, 0)	0 (0, 0)	0 (0, 0)
Taiwan	Specificity	1 (1, 1)	1 (1, 1)	1 (1, 1)	1 (1, 1)
Taiwan	PPV	0 (0, 0)	0 (0, 0)	0 (0, 0)	0 (0, 0)
Taiwan	NPV	0.85 (0.83, 0.87)	0.87 (0.85, 0.89)	0.86 (0.83, 0.88)	0.93 (0.9, 0.97)
Taiwan	F1	0 (0, 0)	0 (0, 0)	0 (0, 0)	0 (0, 0)
Taiwan	Kappa	0 (0, 0)	0 (0, 0)	0 (0, 0)	0 (0, 0)
Würzburg	AUC	0.65 (0.55, 0.75)	0.58 (0.54, 0.62)	0.6 (0.45, 0.75)	0.58 (0.4, 0.76)
Würzburg	Sensitivity	0.13 (0.07, 0.2)	0.1 (-0.02, 0.22)	0.1 (-0.02, 0.22)	0 (0, 0)
Würzburg	Specificity	1 (1, 1)	1 (1, 1)	1 (1, 1)	1 (1, 1)

Würzburg	PPV	0.8 (0.41, 1.19)	0.4 (-0.08, 0.88)	0.4 (-0.08, 0.88)	0 (0, 0)
Würzburg	NPV	0.84 (0.82, 0.85)	0.89 (0.87, 0.9)	0.88 (0.86, 0.89)	0.93 (0.91, 0.95)
Würzburg	F1	0.23 (0.12, 0.34)	0.16 (-0.03, 0.35)	0.16 (-0.03, 0.35)	0 (0, 0)
Würzburg	Kappa	0.2 (0.1, 0.29)[a]	0.15 (-0.03, 0.32)[aa]	0.15 (-0.03, 0.32)[a]	0 (0, 0)
Aggregate Analysis, XGB Classifier					
ALL	AUC	0.56 (0.55, 0.58)	0.58 (0.55, 0.61)	0.59 (0.57, 0.6)	0.5 (0.45, 0.55)
ALL	Sensitivity	0.06 (0.04, 0.08)	0.03 (0.02, 0.03)	0.04 (0.03, 0.06)	0 (0, 0)
ALL	Specificity	0.97 (0.96, 0.98)	0.99 (0.98, 1)	0.99 (0.98, 0.99)	1 (1, 1)
ALL	PPV	0.43 (0.37, 0.49)	0.47 (0.34, 0.59)	0.59 (0.44, 0.73)	0 (0, 0)
ALL	NPV	0.69 (0.69, 0.7)	0.77 (0.77, 0.77)	0.75 (0.74, 0.75)	0.82 (0.82, 0.82)
ALL	F1	0.1 (0.06, 0.13)	0.05 (0.03, 0.06)	0.08 (0.05, 0.11)	0 (0, 0)
ALL	Kappa	0.03 (0.01, 0.05)	0.02 (0.01, 0.03)	0.05 (0.02, 0.07)	0 (-0.01, 0)
Site Level Analysis, XGB Classifier					
Barcelona	AUC	0.38 (0.26, 0.51)	0.52 (0.32, 0.73)	0.45 (0.28, 0.62)	0.45 (0.29, 0.6)
Barcelona	Sensitivity	0.1 (-0.02, 0.22)	0.13 (-0.13, 0.39)	0.07 (-0.06, 0.2)	0 (0, 0)
Barcelona	Specificity	0.83 (0.7, 0.95)	0.95 (0.89, 1.01)	0.88 (0.72, 1.05)	0.93 (0.8, 1.06)
Barcelona	PPV	0.17 (-0.04, 0.37)	0.2 (-0.19, 0.59)	0.05 (-0.05, 0.15)	0 (0, 0)
Barcelona	NPV	0.65 (0.6, 0.71)	0.74 (0.67, 0.81)	0.66 (0.63, 0.68)	0.73 (0.69, 0.76)
Barcelona	F1	0.12 (-0.03, 0.27)	0.16 (-0.15, 0.47)	0.06 (-0.05, 0.17)	0 (0, 0)
Barcelona	Kappa	-0.08 (-0.26, 0.11)	0.09 (-0.24, 0.42)	-0.06 (-0.13, 0.02)	-0.07 (-0.2, 0.06)
Cagliari	AUC	0.51 (0.46, 0.56)	0.41 (0.23, 0.59)	0.43 (0.34, 0.51)	0.55 (0.32, 0.79)
Cagliari	Sensitivity	0.09 (0.09, 0.09)	0 (0, 0)	0 (0, 0)	0 (0, 0)
Cagliari	Specificity	0.85 (0.77, 0.94)	1 (1, 1)	0.99 (0.97, 1.01)	1 (1, 1)
Cagliari	PPV	0.36 (0.04, 0.67)	0 (0, 0)	0 (0, 0)	0 (0, 0)
Cagliari	NPV	0.66 (0.65, 0.68)	0.83 (0.82, 0.84)	0.8 (0.79, 0.81)	0.9 (0.89, 0.9)
Cagliari	F1	0.13 (0.11, 0.15)	0 (0, 0)	0 (0, 0)	0 (0, 0)
Cagliari	Kappa	-0.06 (-0.16, 0.04)	0 (0, 0)	-0.01 (-0.04, 0.01)	0 (0, 0)
Geneva	AUC	0.71 (0.52, 0.89)	0.58 (0.46, 0.7)	0.38 (0.1, 0.67)	0.72 (0.43, 1.01)

Geneva	Sensitivity	0.1 (-0.1, 0.3)	0 (0, 0)	0 (0, 0)	0 (0, 0)
Geneva	Specificity	0.98 (0.93, 1.02)	1 (1, 1)	1 (1, 1)	1 (1, 1)
Geneva	PPV	0.2 (-0.19, 0.59)	0 (0, 0)	0 (0, 0)	0 (0, 0)
Geneva	NPV	0.77 (0.7, 0.83)	0.82 (0.79, 0.84)	0.79 (0.76, 0.82)	0.91 (0.85, 0.98)
Geneva	F1	0.13 (-0.13, 0.39)	0 (0, 0)	0 (0, 0)	0 (0, 0)
Geneva	Kappa	0.09 (-0.17, 0.35)	0 (0, 0)	0 (0, 0)	0 (0, 0)
Halifax	AUC	0.54 (0.49, 0.6)	0.55 (0.52, 0.59)	0.56 (0.48, 0.65)	0.6 (0.55, 0.66)
Halifax	Sensitivity	0.43 (0.37, 0.5)	0.32 (0.27, 0.37)	0.39 (0.29, 0.49)	0.12 (0.02, 0.23)
Halifax	Specificity	0.64 (0.58, 0.7)	0.8 (0.76, 0.83)	0.68 (0.61, 0.76)	0.97 (0.95, 0.98)
Halifax	PPV	0.52 (0.48, 0.56)	0.54 (0.53, 0.55)	0.5 (0.38, 0.61)	0.43 (0.09, 0.78)
Halifax	NPV	0.56 (0.53, 0.58)	0.62 (0.61, 0.63)	0.58 (0.53, 0.63)	0.69 (0.66, 0.72)
Halifax	F1	0.47 (0.42, 0.52)	0.4 (0.36, 0.44)	0.44 (0.33, 0.54)	0.19 (0.03, 0.35)
Halifax	Kappa	0.07 (0.01, 0.13)	0.13 (0.1, 0.15)	0.07 (-0.07, 0.22)	0.11 (0, 0.23)
Japan	AUC	0.49 (0.36, 0.62)	0.62 (0.47, 0.78)	0.68 (0.62, 0.74)	0.62 (0.47, 0.78)
Japan	Sensitivity	0 (0, 0)	0 (0, 0)	0.04 (-0.04, 0.12)	0.07 (-0.06, 0.2)
Japan	Specificity	0.99 (0.97, 1.01)	0.99 (0.97, 1.01)	0.96 (0.93, 0.99)	0.98 (0.95, 1.02)
Japan	PPV	0 (0, 0)	0 (0, 0)	0.2 (-0.19, 0.59)	0.2 (-0.19, 0.59)
Japan	NPV	0.74 (0.73, 0.75)	0.78 (0.77, 0.78)	0.75 (0.73, 0.78)	0.76 (0.71, 0.8)
Japan	F1	0 (0, 0)	0 (0, 0)	0.07 (-0.06, 0.2)	0.1 (-0.1, 0.3)
Japan	Kappa	-0.02 (-0.05, 0.01)	-0.02 (-0.05, 0.02)	0 (-0.14, 0.14)	0.06 (-0.13, 0.25)
Mayo Clinic	AUC	0.71 (0.57, 0.86)	0.68 (0.51, 0.85)	0.58 (0.42, 0.75)	0.06 (-0.06, 0.18)
Mayo Clinic	Sensitivity	0.04 (-0.04, 0.12)	0 (0, 0)	0.07 (-0.06, 0.2)	0 (0, 0)
Mayo Clinic	Specificity	0.94 (0.88, 0.99)	1 (1, 1)	0.96 (0.91, 1.01)	1 (1, 1)
Mayo Clinic	PPV	0.1 (-0.1, 0.3)	0 (0, 0)	0.2 (-0.19, 0.59)	0 (0, 0)
Mayo Clinic	NPV	0.73 (0.71, 0.76)	0.82 (0.8, 0.84)	0.79 (0.75, 0.82)	0.91 (0.87, 0.96)
Mayo Clinic	F1	0.06 (-0.05, 0.17)	0 (0, 0)	0.1 (-0.1, 0.3)	0 (0, 0)
Mayo Clinic	Kappa	-0.03 (-0.14, 0.08)	0 (0, 0)	0.03 (-0.17, 0.24)	0 (0, 0)
Paris	AUC	0.47 (0.4, 0.54)	0.42 (0.39, 0.46)	0.57 (0.48, 0.65)	0.45 (0.23, 0.67)
Paris	Sensitivity	0 (0, 0)	0 (0, 0)	0 (0, 0)	0 (0, 0)

Paris	Specificity	1 (1, 1)	1 (1, 1)	1 (1, 1)	1 (1, 1)
Paris	PPV	0 (0, 0)	0 (0, 0)	0 (0, 0)	0 (0, 0)
Paris	NPV	0.8 (0.79, 0.8)	0.9 (0.89, 0.91)	0.88 (0.86, 0.89)	0.94 (0.92, 0.96)
Paris	F1	0 (0, 0)	0 (0, 0)	0 (0, 0)	0 (0, 0)
Paris	Kappa	0 (0, 0)	0 (0, 0)	0 (0, 0)	0 (0, 0)
Poznan	AUC	0.65 (0.54, 0.76)	0.62 (0.47, 0.76)	0.41 (0.3, 0.53)	0.69 (0.59, 0.8)
Poznan	Sensitivity	0.47 (0.3, 0.64)	0.54 (0.32, 0.76)	0.76 (0.66, 0.85)	0.85 (0.7, 0.99)
Poznan	Specificity	0.61 (0.38, 0.83)	0.59 (0.49, 0.68)	0.12 (0.02, 0.22)	0.3 (0.04, 0.56)
Poznan	PPV	0.6 (0.51, 0.69)	0.52 (0.43, 0.6)	0.56 (0.52, 0.6)	0.69 (0.56, 0.82)
Poznan	NPV	0.51 (0.49, 0.52)	0.62 (0.48, 0.75)	0.23 (0.04, 0.43)	0.53 (0.09, 0.98)
Poznan	F1	0.49 (0.4, 0.59)	0.51 (0.37, 0.66)	0.64 (0.58, 0.7)	0.76 (0.63, 0.89)
Poznan	Kappa	0.08 (0.02, 0.14)	0.12 (-0.08, 0.32)	-0.13 (-0.29, 0.02)	0.16 (-0.24, 0.57)
Romania	AUC	0.44 (0.33, 0.55)	0.6 (0.44, 0.76)	0.67 (0.58, 0.76)	0.4 (0.13, 0.67)
Romania	Sensitivity	0 (0, 0)	0 (0, 0)	0 (0, 0)	0 (0, 0)
Romania	Specificity	1 (1, 1)	1 (1, 1)	1 (1, 1)	1 (1, 1)
Romania	PPV	0 (0, 0)	0 (0, 0)	0 (0, 0)	0 (0, 0)
Romania	NPV	0.77 (0.76, 0.79)	0.9 (0.88, 0.92)	0.89 (0.87, 0.91)	0.96 (0.93, 0.98)
Romania	F1	0 (0, 0)	0 (0, 0)	0 (0, 0)	0 (0, 0)
Romania	Kappa	0 (0, 0)	0 (0, 0)	0 (0, 0)	0 (0, 0)
San Diego	AUC	0.51 (0.48, 0.54)	0.2 (0.12, 0.28)	0.4 (0.32, 0.48)	0.47 (0.29, 0.64)
San Diego	Sensitivity	0 (0, 0)	0 (0, 0)	0 (0, 0)	0 (0, 0)
San Diego	Specificity	1 (1, 1)	1 (1, 1)	1 (1, 1)	1 (1, 1)
San Diego	PPV	0 (0, 0)	0 (0, 0)	0 (0, 0)	0 (0, 0)
San Diego	NPV	0.88 (0.87, 0.89)	0.95 (0.95, 0.95)	0.94 (0.94, 0.94)	0.96 (0.96, 0.96)
San Diego	F1	0 (0, 0)	0 (0, 0)	0 (0, 0)	0 (0, 0)
San Diego	Kappa	0 (0, 0)	0 (0, 0)	0 (0, 0)	0 (0, 0)
Sweden	AUC	0.4 (0.31, 0.49)	0.59 (0.53, 0.65)	0.42 (0.35, 0.5)	0.53 (0.43, 0.62)
Sweden	Sensitivity	0.31 (0.25, 0.37)	0.16 (0.05, 0.27)	0.16 (0.08, 0.24)	0.07 (0.01, 0.13)
Sweden	Specificity	0.58 (0.46, 0.69)	0.92 (0.88, 0.96)	0.79 (0.73, 0.86)	0.94 (0.9, 0.98)

Sweden	PPV	0.42 (0.31, 0.53)	0.42 (0.15, 0.7)	0.35 (0.16, 0.54)	0.27 (0.05, 0.49)
Sweden	NPV	0.47 (0.41, 0.53)	0.66 (0.63, 0.69)	0.57 (0.54, 0.6)	0.68 (0.66, 0.71)
Sweden	F1	0.36 (0.28, 0.43)	0.23 (0.07, 0.38)	0.21 (0.11, 0.32)	0.11 (0.02, 0.2)
Sweden	Kappa	-0.11 (-0.27, 0.04)	0.09 (-0.04, 0.21)	-0.05 (-0.19, 0.08)	0.01 (-0.07, 0.09)
Sydney	AUC	0.57 (0.35, 0.79)	0.22 (0.12, 0.31)	0.44 (0.38, 0.5)	0.46 (0.22, 0.7)
Sydney	Sensitivity	0 (0, 0)	0 (0, 0)	0 (0, 0)	0 (0, 0)
Sydney	Specificity	0.98 (0.93, 1.02)	1 (1, 1)	1 (1, 1)	1 (1, 1)
Sydney	PPV	0 (0, 0)	0 (0, 0)	0 (0, 0)	0 (0, 0)
Sydney	NPV	0.79 (0.76, 0.81)	0.82 (0.79, 0.84)	0.79 (0.77, 0.82)	0.87 (0.85, 0.9)
Sydney	F1	0 (0, 0)	0 (0, 0)	0 (0, 0)	0 (0, 0)
Sydney	Kappa	-0.03 (-0.08, 0.03)	0 (0, 0)	0 (0, 0)	0 (0, 0)
Taiwan	AUC	0.68 (0.44, 0.91)	0.55 (0.48, 0.62)	0.51 (0.41, 0.61)	0.66 (0.53, 0.79)
Taiwan	Sensitivity	0.07 (-0.06, 0.2)	0 (0, 0)	0 (0, 0)	0 (0, 0)
Taiwan	Specificity	1 (1, 1)	1 (1, 1)	1 (1, 1)	1 (1, 1)
Taiwan	PPV	0.2 (-0.19, 0.59)	0 (0, 0)	0 (0, 0)	0 (0, 0)
Taiwan	NPV	0.86 (0.84, 0.88)	0.87 (0.85, 0.89)	0.86 (0.83, 0.88)	0.93 (0.9, 0.97)
Taiwan	F1	0.1 (-0.1, 0.3)	0 (0, 0)	0 (0, 0)	0 (0, 0)
Taiwan	Kappa	0.09 (-0.09, 0.27)	0 (0, 0)	0 (0, 0)	0 (0, 0)
Würzburg	AUC	0.56 (0.52, 0.61)	0.54 (0.44, 0.64)	0.5 (0.4, 0.59)	0.28 (0.12, 0.45)
Würzburg	Sensitivity	0.13 (0.07, 0.2)	0.1 (-0.02, 0.22)	0.1 (-0.02, 0.22)	0 (0, 0)
Würzburg	Specificity	0.99 (0.98, 1.01)	0.98 (0.95, 1.01)	0.99 (0.98, 1.01)	1 (1, 1)
Würzburg	PPV	0.7 (0.31, 1.09)	0.4 (-0.08, 0.88)	0.4 (-0.08, 0.88)	0 (0, 0)
Würzburg	NPV	0.84 (0.82, 0.85)	0.89 (0.87, 0.9)	0.88 (0.86, 0.89)	0.93 (0.91, 0.95)
Würzburg	F1	0.22 (0.11, 0.33)	0.16 (-0.03, 0.35)	0.16 (-0.03, 0.35)	0 (0, 0)
Würzburg	Kappa	0.18 (0.09, 0.28)[a]	0.13 (-0.06, 0.32)	0.13 (-0.05, 0.32)	0 (0, 0)
a - Kappa value was found to have a p-value less than 0.01 in comparison to the null classifier.					

Classification Analysis Stratified by Follow-Up Methodology

The methodology for this analysis is described in the main text. Briefly, we split the dataset into two subsets: (A) one including only those sites who followed subjects prospectively, and (B) one including sites that did *not* follow subjects prospectively. The aggregate, leave-one-site-out, and predict-one-site-out analyses were repeated *within* each of these conditions, respectively. Our primary outcome metric in this analysis was Cohen's kappa, whose mean and 95% confidence intervals across folds were compared in the aggregate analysis analysis-wise between models trained on the prospective and retrospective datasets. Since the leave-one-site-out analyses do not have direct comparators across the prospective and retrospective datasets, we estimated the "statistical significance" of Cohen's kappa results using the p-value simulation procedure described below.

Classification Results

Table S11

Table S11: This table displays the results for both the LR and XGB classifiers in the aggregate, leave one site out, and predict one site out analyses performed separately on the prospectively collected and non-prospectively collected samples. Asterisks in the rows that represent the Kappa statistic signify a p-value less than 0.01 in comparison to the simulated null classifier. *Abbreviations*: all sites (ALL; i.e. aggregate analysis), area under the receiver operating characteristic curve (AUC), positive predictive value (PPV), negative predictive value (NPV), F-1 score (F1).

Model	Site	Accuracy	AUC	Sensitivity	Specificity	PPV	NPV	F1	Kappa	Follow Up
Aggregate Analysis										
LR	ALL	0.66 (0.64, 0.67)	0.61 (0.58, 0.65)	0.11 (0.06, 0.15)	0.97 (0.95, 0.98)	0.66 (0.55, 0.77)	0.66 (0.65, 0.67)	0.18 (0.11, 0.25)	0.09 (0.04, 0.14)	Prospective
LR	ALL	0.76 (0.76, 0.76)	0.59 (0.55, 0.63)	0 (0, 0.01)	1 (1, 1)	0.2 (0, 0.59)	0.76 (0.76, 0.76)	0.01 (0, 0.02)	0 (-0.01, 0.01)	Retrospective
XGB	ALL	0.65 (0.63, 0.67)	0.56 (0.52, 0.6)	0.16 (0.1, 0.22)	0.92 (0.91, 0.93)	0.51 (0.42, 0.6)	0.66 (0.65, 0.68)	0.24 (0.16, 0.32)	0.09 (0.02, 0.16)	Prospective
XGB	ALL	0.76 (0.75, 0.77)	0.53 (0.49, 0.57)	0.01 (0, 0.02)	1 (0.99, 1)	0.5 (0.06, 0.94)	0.76 (0.76, 0.76)	0.02 (0, 0.05)	0.01 (-0.01, 0.03)	Retrospective
Leave One Site Out Analysis										
LR	Barcelona	0.64 (0.61, 0.66)	0.59 (0.55, 0.63)	0.1 (0.05, 0.14)	0.95 (0.93, 0.98)	0.53 (0.35, 0.72)	0.64 (0.63, 0.66)	0.16 (0.09, 0.23)	0.06 (-0.01, 0.12)	Prospective
LR	Cagliari	0.64 (0.62, 0.65)	0.62 (0.6, 0.65)	0.11 (0.09, 0.12)	0.96 (0.94, 0.98)	0.66 (0.53, 0.78)	0.64 (0.63, 0.64)	0.19 (0.16, 0.21)	0.08 (0.05, 0.11)	Prospective
LR	Halifax	0.7 (0.7, 0.71)	0.57 (0.54, 0.59)	0 (0, 0)	1 (1, 1)	0 (0, 0)	0.7 (0.7, 0.71)	0 (0, 0)	0 (0, 0)	Prospective

LR	Poznan	0.67 (0.65, 0.68)	0.63 (0.57, 0.69)	0.06 (0.02, 0.1)	0.99 (0.98, 0.99)	0.63 (0.5, 0.77)	0.67 (0.66, 0.68)	0.11 (0.04, 0.17)	0.06 (0.01, 0.11)	Prospective
LR	Romania	0.62 (0.57, 0.67)	0.62 (0.54, 0.7)	0.18 (0.11, 0.25)	0.9 (0.86, 0.95)	0.55 (0.39, 0.7)	0.63 (0.6, 0.66)	0.27 (0.17, 0.36)	0.09 (-0.02, 0.21)[a]	Prospective
LR	Geneva	0.76 (0.76, 0.76)	0.61 (0.59, 0.64)	0.01 (0.01, 0.02)	1 (1, 1)	0.6 (0.23, 0.97)	0.76 (0.76, 0.76)	0.03 (0.01, 0.04)	0.02 (0.01, 0.03)	Retrospective
LR	Japan	0.76 (0.76, 0.77)	0.6 (0.58, 0.63)	0.02 (0, 0.03)	1 (0.99, 1)	0.5 (0.06, 0.94)	0.76 (0.76, 0.77)	0.03 (0, 0.06)	0.02 (0, 0.04)	Retrospective
LR	Mayo Clinic	0.76 (0.76, 0.76)	0.58 (0.56, 0.61)	0.01 (0.01, 0.02)	1 (1, 1)	0.6 (0.23, 0.97)	0.76 (0.76, 0.76)	0.03 (0.01, 0.04)	0.02 (0.01, 0.03)	Retrospective
LR	Paris	0.75 (0.75, 0.75)	0.61 (0.57, 0.64)	0.01 (0, 0.02)	1 (1, 1)	0.3 (0, 0.69)	0.75 (0.75, 0.75)	0.01 (0, 0.03)	0.01 (0, 0.02)	Retrospective
LR	San Diego	0.74 (0.73, 0.74)	0.6 (0.56, 0.63)	0.02 (0.01, 0.03)	1 (0.99, 1)	0.53 (0.22, 0.85)	0.74 (0.74, 0.74)	0.03 (0.01, 0.05)	0.02 (0.01, 0.03)	Retrospective
LR	Sweden	0.83 (0.82, 0.83)	0.51 (0.46, 0.57)	0.02 (0, 0.04)	1 (1, 1)	0.6 (0.12, 1)	0.83 (0.82, 0.83)	0.04 (0, 0.08)	0.03 (0, 0.07)	Retrospective
LR	Sydney	0.76 (0.76, 0.76)	0.62 (0.59, 0.66)	0.02 (0.02, 0.02)	1 (1, 1)	0.9 (0.7, 1)	0.76 (0.76, 0.76)	0.03 (0.03, 0.03)	0.02 (0.02, 0.03)	Retrospective
LR	Taiwan	0.76 (0.76, 0.76)	0.62 (0.6, 0.64)	0.02 (0.01, 0.03)	1 (1, 1)	1 (1, 1)	0.76 (0.76, 0.76)	0.04 (0.03, 0.05)	0.03 (0.02, 0.04)	Retrospective
LR	Würzburg	0.75 (0.75, 0.75)	0.59 (0.55, 0.63)	0.01 (0, 0.02)	1 (0.99, 1)	0.17 (0, 0.37)	0.75 (0.75, 0.75)	0.01 (0, 0.03)	0.01 (0, 0.01)	Retrospective
XGB	Barcelona	0.63 (0.59, 0.67)	0.56 (0.51, 0.61)	0.15 (0.13, 0.17)	0.9 (0.86, 0.95)	0.52 (0.35, 0.68)	0.65 (0.63, 0.66)	0.23 (0.19, 0.28)	0.07 (-0.01, 0.15)	Prospective
XGB	Cagliari	0.62 (0.61, 0.62)	0.54 (0.49, 0.58)	0.19 (0.16, 0.22)	0.88 (0.87, 0.89)	0.49 (0.47, 0.51)	0.64 (0.63, 0.64)	0.27 (0.24, 0.31)	0.08 (0.06, 0.1)	Prospective
XGB	Halifax	0.7 (0.68, 0.72)	0.52 (0.47, 0.58)	0.07 (0, 0.14)	0.96 (0.95, 0.98)	0.32 (0.06, 0.58)	0.71 (0.69, 0.73)	0.1 (0, 0.22)	0.04 (-0.05, 0.12)	Prospective
XGB	Poznan	0.65 (0.63, 0.66)	0.53 (0.5, 0.56)	0.12 (0.07, 0.17)	0.92 (0.9, 0.94)	0.43 (0.32, 0.55)	0.67 (0.66, 0.68)	0.19 (0.12, 0.25)	0.05 (-0.01, 0.11)	Prospective
XGB	Romania	0.57 (0.52, 0.63)	0.51 (0.43, 0.59)	0.17 (0.12, 0.22)	0.83 (0.77, 0.89)	0.41 (0.28, 0.54)	0.61 (0.58, 0.64)	0.24 (0.17, 0.31)	0 (-0.11, 0.11)	Prospective
XGB	Geneva	0.76 (0.76, 0.77)	0.55 (0.51, 0.6)	0.02 (0.01, 0.03)	0.99 (0.99, 1)	0.65 (0.36, 0.93)	0.76 (0.76, 0.76)	0.04 (0.03, 0.06)	0.02 (0.01, 0.04)	Retrospective
XGB	Japan	0.76 (0.76, 0.77)	0.55 (0.51, 0.59)	0.03 (0.01, 0.04)	1 (0.99, 1)	0.67 (0.31, 1)	0.76 (0.76, 0.77)	0.05 (0.02, 0.09)	0.04 (0.01, 0.06)	Retrospective

XGB	Mayo Clinic	0.76 (0.76, 0.77)	0.56 (0.53, 0.6)	0.03 (0.02, 0.03)	1 (0.99, 1)	0.73 (0.51, 0.95)	0.76 (0.76, 0.76)	0.05 (0.04, 0.07)	0.03 (0.02, 0.04)	Retrospective
XGB	Paris	0.75 (0.74, 0.75)	0.56 (0.53, 0.59)	0.03 (0.01, 0.04)	0.99 (0.98, 1)	0.46 (0.19, 0.72)	0.75 (0.75, 0.76)	0.05 (0.03, 0.08)	0.02 (0, 0.05)	Retrospective
XGB	San Diego	0.73 (0.72, 0.74)	0.53 (0.48, 0.57)	0.03 (0.02, 0.04)	0.99 (0.98, 1)	0.51 (0.24, 0.77)	0.74 (0.73, 0.74)	0.05 (0.04, 0.07)	0.02 (0, 0.04)	Retrospective
XGB	Sweden	0.82 (0.82, 0.83)	0.49 (0.44, 0.53)	0.02 (0, 0.04)	1 (0.99, 1)	0.43 (0.05, 0.81)	0.83 (0.82, 0.83)	0.04 (0, 0.08)	0.03 (0, 0.06)	Retrospective
XGB	Sydney	0.76 (0.76, 0.76)	0.54 (0.5, 0.57)	0.02 (0.01, 0.03)	1 (1, 1)	0.8 (0.41, 1)	0.76 (0.76, 0.76)	0.03 (0.01, 0.05)	0.02 (0.01, 0.04)	Retrospective
XGB	Taiwan	0.75 (0.75, 0.76)	0.52 (0.51, 0.53)	0.02 (0.01, 0.03)	0.99 (0.98, 1)	0.47 (0.15, 0.78)	0.75 (0.75, 0.76)	0.03 (0.01, 0.05)	0.01 (0, 0.02)	Retrospective
XGB	Würzburg	0.75 (0.74, 0.75)	0.56 (0.51, 0.6)	0.02 (0, 0.04)	0.99 (0.98, 0.99)	0.36 (0.11, 0.61)	0.75 (0.75, 0.76)	0.04 (0.01, 0.07)	0.01 (-0.02, 0.04)	Retrospective
Predict One Site Out Analysis										
LR	Barcelona	0.72	0.61	0.00	0.98	0.00	0.73	0.00	-0.03	Prospective
LR	Cagliari	0.72	0.52	0.00	1.00	0.00	0.72	0.00	0.00	Prospective
LR	Halifax	0.55	0.55	0.00	1.00	0.00	0.55	0.00	0.00	Prospective
LR	Poznan	0.51	0.53	0.09	0.90	0.44	0.51	0.14	-0.02	Prospective
LR	Romania	0.74	0.54	0.13	0.91	0.27	0.80	0.17	0.04	Prospective
LR	Geneva	0.77	0.57	0.00	1.00	0.00	0.77	0.00	0.00	Retrospective
LR	Japan	0.76	0.51	0.00	1.00	0.00	0.76	0.00	0.00	Retrospective
LR	Mayo Clinic	0.77	0.65	0.00	1.00	0.00	0.77	0.00	0.00	Retrospective
LR	Paris	0.82	0.54	0.00	1.00	0.00	0.82	0.00	0.00	Retrospective
LR	San Diego	0.89	0.50	0.00	1.00	0.00	0.89	0.00	0.00	Retrospective
LR	Sweden	0.55	0.49	0.00	1.00	0.00	0.55	0.00	0.00	Retrospective
LR	Sydney	0.79	0.35	0.00	1.00	0.00	0.79	0.00	0.00	Retrospective
LR	Taiwan	0.86	0.44	0.00	1.00	0.00	0.86	0.00	0.00	Retrospective

LR	Würzburg	0.83	0.50	0.00	1.00	0.00	0.83	0.00	0.00	Retrospective
XGB	Barcelona	0.68	0.56	0.05	0.91	0.17	0.72	0.08	-0.05	Prospective
XGB	Cagliari	0.69	0.45	0.04	0.95	0.22	0.72	0.06	-0.02	Prospective
XGB	Halifax	0.54	0.49	0.03	0.95	0.33	0.54	0.06	-0.02	Prospective
XGB	Poznan	0.47	0.48	0.09	0.84	0.33	0.49	0.14	-0.08	Prospective
XGB	Romania	0.71	0.60	0.22	0.84	0.27	0.80	0.24	0.06	Prospective
XGB	Geneva	0.77	0.39	0.00	1.00	0.00	0.77	0.00	0.00	Retrospective
XGB	Japan	0.76	0.49	0.00	1.00	0.00	0.76	0.00	0.00	Retrospective
XGB	Mayo Clinic	0.77	0.53	0.00	1.00	0.00	0.77	0.00	0.00	Retrospective
XGB	Paris	0.81	0.52	0.00	0.99	0.00	0.82	0.00	-0.01	Retrospective
XGB	San Diego	0.89	0.52	0.00	1.00	0.00	0.89	0.00	0.00	Retrospective
XGB	Sweden	0.55	0.49	0.00	1.00	0.00	0.55	0.00	0.00	Retrospective
XGB	Sydney	0.79	0.42	0.00	1.00	0.00	0.79	0.00	0.00	Retrospective
XGB	Taiwan	0.86	0.47	0.00	1.00	0.00	0.86	0.00	0.00	Retrospective
XGB	Würzburg	0.82	0.51	0.00	0.99	0.00	0.83	0.00	-0.01	Retrospective
a - Kappa value was found to have a p-value less than 0.01 in comparison to the null classifier.										

Gene Set Analyses

Table S12

Table S12: This table displays results from the PANTHER statistical overrepresentation test using the logistic regression classifier for the feature importance metric on the aggregate prospectively followed sample. The selected sample of variant associated genes were those for which the regression coefficients had the same sign across all five cross validation folds and for which the absolute value of the median was in the top quartile of those with the same sign across folds.						
Functional Class	N Ref	N Obs	N Exp	Factor	p (Raw)	FDR
GO Cellular Component Complete						
presynaptic membrane (GO:0042734)	103	52	26.09	1.99	1.05E-04	2.23E-02

postsynaptic membrane (GO:0045211)	190	84	48.12	1.75	3.30E-05	9.37E-03
synaptic membrane (GO:0097060)	254	111	64.33	1.73	2.77E-06	4.72E-03
synapse part (GO:0044456)	495	175	125.38	1.4	1.18E-04	2.23E-02
synapse (GO:0045202)	614	217	155.52	1.4	1.64E-05	6.99E-03
cell junction (GO:0030054)	642	219	162.61	1.35	1.04E-04	2.52E-02
plasma membrane region (GO:0098590)	643	216	162.86	1.33	2.13E-04	3.62E-02
plasma membrane part (GO:0044459)	1413	448	357.89	1.25	5.29E-06	4.51E-03
plasma membrane (GO:0005886)	2422	714	613.46	1.16	1.43E-05	8.10E-03
cell periphery (GO:0071944)	2478	727	627.64	1.16	2.02E-05	6.88E-03
GO Biological Process Complete						
generation of neurons (GO:0048699)	747	256	189.21	1.35	1.47E-05	3.85E-02
neurogenesis (GO:0022008)	794	272	201.11	1.35	8.00E-06	3.50E-02
nervous system development (GO:0007399)	1115	372	282.41	1.32	7.99E-07	1.05E-02
cell differentiation (GO:0030154)	1627	499	412.1	1.21	2.54E-05	4.16E-02
system development (GO:0048731)	1994	610	505.05	1.21	2.09E-06	1.37E-02
multicellular organism development (GO:0007275)	2242	664	567.87	1.17	2.32E-05	4.35E-02
anatomical structure development (GO:0048856)	2394	709	606.37	1.17	9.26E-06	3.04E-02
developmental process (GO:0032502)	2519	739	638.03	1.16	1.51E-05	3.31E-02

Table S13

Table S13: This table displays the top result of the statistical overrepresentation test for the gene ontology cellular component analysis on the aggregated prospective sample. The presynaptic membrane was the function annotation for which we found the highest enrichment factor in comparison to the reference set.					
Gene ID	Gene	PANTHER Protein Class	Gene ID	Gene	
SLC6A5	Sodium- and chloride-dependent glycine transporter 2	cation transporter(PC00068)	ERBB4	Receptor tyrosine-protein kinase erbB-4	
CTNNA2	Catenin alpha-2	cell adhesion molecule(PC00069);non-motor actin binding protein(PC00165)	ADCY8	Adenylate cyclase type 8	
IL31RA	Interleukin-31 receptor subunit alpha	cytokine(PC00083);defense/immunity protein(PC00090)	NTNG1	Netrin-G1	extracellular matrix linker protein(PC00101);protease inhibitor(PC00191);receptor (PC00197)
CAPS2	Calcium-dependent secretion activator 2	calcium-binding protein(PC00060)	SLC6A11	Sodium- and chloride-dependent GABA transporter 3	cation transporter(PC00068)
GPM6A	Neuronal membrane glycoprotein M6-a	myelin protein(PC00161)	RIMS2	Regulating synaptic membrane exocytosis protein 2	G-protein modulator(PC00022)
CHRM3	Muscarinic acetylcholine receptor M3	G-protein coupled receptor(PC00021)	GRM8	Metabotropic glutamate receptor 8	G-protein coupled receptor(PC00021)
PSEN2	Presenilin-2	aspartic protease(PC00053);calcium-binding protein(PC00060);membrane-bound signaling molecule(PC00152)	KCNH1	Potassium voltage-gated channel subfamily H member 1	
DGKI	Diacylglycerol kinase iota	kinase(PC00137)	GRIK2	Glutamate receptor ionotropic, kainate 5	
KCNA2	Potassium voltage-gated channel subfamily A member 2		ATP2B4,ATP2B2	Plasma membrane calcium-transporting ATPase 4	cation transporter(PC00068);hydrolase(PC00121);ion channel(PC00133)
CNTN1	Contactin-1		CNTN6	Contactin-6	transmembrane receptor regulatory/adaptor protein(PC00226)
P2RX3	P2X purinoceptor 3		DRD2	D(2) dopamine receptor	G-protein coupled

					receptor(PC00021)
HIP1	Huntingtin-interacting protein 1	non-motor actin binding protein(PC00165)	DENND1A	DENN domain-containing protein 1A	
PICALM	Phosphatidylinositol-binding clathrin assembly protein	vesicle coat protein(PC00235)	ERC2	ERC protein 2	G-protein modulator(PC00022);membrane traffic protein(PC00150)
KCNC2	Potassium voltage-gated channel subfamily C member 2		GRIK2	Glutamate receptor ionotropic, kainate 2	
SLC6A3	Sodium-dependent dopamine transporter	cation transporter(PC00068)	SYT1	Synaptotagmin-1	membrane trafficking regulatory protein(PC00151)
GAD2	Glutamate decarboxylase 2	decarboxylase(PC00089)	GRIK4	Glutamate receptor ionotropic, kainate 4	
KCTD16	BTB/POZ domain-containing protein KCTD16	enzyme modulator(PC00095)	SCRIB	Protein scribble homolog	
ADRA1A	Alpha-1A adrenergic receptor	G-protein coupled receptor(PC00021)	CNTN5	Contactin-5	
RIMS1	Regulating synaptic membrane exocytosis protein 1	G-protein modulator(PC00022)	SLC1A2	Excitatory amino acid transporter 2	cation transporter(PC00068)
RIMS3	Regulating synaptic membrane exocytosis protein 3	G-protein modulator(PC00022)	CACNA1D	Voltage-dependent L-type calcium channel subunit alpha-1D	
APBA1	Amyloid-beta A4 precursor protein-binding family A member 1	membrane trafficking regulatory protein(PC00151)	HTT	Sodium-dependent serotonin transporter	cation transporter(PC00068)
EPHB2	Ephrin type-B receptor 2		NRXN1	Neurexin-1	
NRG1	Pro-neuregulin-1, membrane-bound isoform	growth factor(PC00112)	GRM7	Metabotropic glutamate receptor 7	G-protein coupled receptor(PC00021)
SNCAIP	Synphilin-1		CHRM2	Muscarinic acetylcholine receptor M2	G-protein coupled receptor(PC00021)
OPRM1	Mu-type opioid receptor	G-protein coupled receptor(PC00021)	GRM3	Metabotropic glutamate receptor 3	G-protein coupled receptor(PC00021)
OPRD1	Delta-type opioid receptor	G-protein coupled receptor(PC00021)	SLC6A5,NAT1	Sodium-dependent noradrenaline transporter	cation transporter(PC00068)

Table S14

Table S14: This table displays the top result of the statistical overrepresentation test for the gene ontology biological component analysis on the aggregated prospective sample. The generation of neurons class was the function annotation for which we found the highest enrichment factor in comparison to the reference set.					
Gene ID	Gene	PANTHER Protein Class	Gene ID	Gene	
PTPRG	Receptor-type tyrosine-protein phosphatase gamma	protein phosphatase(PC00195);receptor(PC00197)	SEMA4F	Semaphorin-4F	membrane-bound signaling molecule(PC00152)
DPYSL2	Dihydropyrimidinase-related protein 2;DPYSL2;ortholog	metalloprotease(PC00153)	ETV1	ETS translocation variant 1	nucleic acid binding(PC00171);signaling molecule(PC00207);winged helix/forkhead transcription factor(PC00246)
NOS1	Nitric oxide synthase, brain		EFNA5	Ephrin-A5	membrane-bound signaling molecule(PC00152)
ABL1	Tyrosine-protein kinase ABL1		MET	Hepatocyte growth factor receptor	
ADGRF1	Adhesion G-protein coupled receptor F1		NTNG1	Netrin-G1	extracellular matrix linker protein(PC00101);protease inhibitor(PC00191);receptor(PC00197)
NOS1	Nanos homolog 1	nuclease(PC00170)	SORL1	Sortilin-related receptor	receptor(PC00197);transporter(PC00227)
PTPRD	Receptor-type tyrosine-protein phosphatase delta	protein phosphatase(PC00195);receptor(PC00197)	TGFBR1	TGF-beta receptor type-1	TGF-beta receptor(PC00035);serine/threonine protein kinase receptor(PC00205)
CTNNA2	Catenin alpha-2	cell adhesion molecule(PC00069);non-motor actin binding protein(PC00165)	THRB	Prothrombin	serine protease(PC00203)
DNMT3A	DNA (cytosine-5)-methyltransferase 3A		MEIS1	Homeobox protein Meis1	DNA-directed RNA polymerase(PC00019);homeodomain transcription factor(PC00119)
SPEN	Msx2-interacting protein		ABI1	Abl interactor 1	G-protein modulator(PC00022)
TNFRSF21	Tumor necrosis factor receptor superfamily member 21		LRRC4C	Leucine-rich repeat-containing protein 4C	
DUSP10	Dual specificity protein phosphatase 10		MME	Neprilysin	metalloprotease(PC00153)
POMGNT2	Protein O-linked-mannose beta-1,4-N-acetylglucosaminyltransferase 2	glycosyltransferase(PC00111)	VLDLR	Very low-density lipoprotein receptor	

PLXNC1	Plexin-C1		ADGRB1	Adhesion G protein-coupled receptor B1	G-protein coupled receptor(PC00021);antibacterial response protein(PC00051);protease(PC00190)
KDM4C	Lysine-specific demethylase 4C	zinc finger transcription factor(PC00244)	MACF1	Microtubule-actin cross-linking factor 1, isoforms 1/2/3/5	intermediate filament binding protein(PC00130)
PIK3R1	Phosphatidylinositol 3-kinase regulatory subunit alpha	kinase modulator(PC00140)	MTCH1	Mitochondrial carrier homolog 1	mitochondrial carrier protein(PC00158);transfer/carrier protein(PC00219)
GAP43	Neuromodulin		LAMB1	Laminin subunit beta-1	extracellular matrix linker protein(PC00101);protease inhibitor(PC00191);receptor(PC00197)
ISPD	D-ribitol-5-phosphate cytidylyltransferase		SECISBP2	Selenocysteine insertion sequence-binding protein 2	
GLI2	Zinc finger protein GLI2		FAM19A1	Protein FAM19A1	
LAMA2	Laminin subunit alpha-2	extracellular matrix linker protein(PC00101);protease inhibitor(PC00191);receptor(PC00197)	ITGA1	Integrin alpha-1	
TMC1	Transmembrane channel-like protein 1		CYFIP2	Cytoplasmic FMR1-interacting protein 2	G-protein modulator(PC00022)
GPM6A	Neuronal membrane glycoprotein M6-a	myelin protein(PC00161)	LPAR3	Lysophosphatidic acid receptor 3	G-protein coupled receptor(PC00021)
SEMA5B	Semaphorin-5B		SMARCC2	SWI/SNF complex subunit SMARCC2	chromatin/chromatin-binding protein(PC00077);metalloprotease(PC00153);transcription cofactor(PC00217)
KANK1	KN motif and ankyrin repeat domain-containing protein 1		RIMS2	Regulating synaptic membrane exocytosis protein 2	G-protein modulator(PC00022)
PTK2	Focal adhesion kinase 1		RIPOR2	Rho family-interacting cell polarization regulator 2	
PHACTR1	Phosphatase and actin regulator 1	phosphatase modulator(PC00184)	ZHX2	Zinc fingers and homeoboxes protein 2	homeodomain transcription factor(PC00119);nucleic acid binding(PC00171);zinc finger transcription factor(PC00244)
NTM	Neurotrimin		RARB	Retinoic acid receptor beta	C4 zinc finger nuclear receptor(PC00169);nucleic acid binding(PC00171);receptor(PC00197)

ESRP1	Epithelial splicing regulatory protein 1	ribosomal protein(PC00202)	PRKN	E3 ubiquitin-protein ligase parkin	ubiquitin-protein ligase(PC00234)
LRRC7	Leucine-rich repeat-containing protein 7		EPHB1	Ephrin type-B receptor 1	
HDAC9	Histone deacetylase 9		FOXO6	Forkhead box protein O6	
NCKAP1L	Nck-associated protein 1-like		SLC1A3	Excitatory amino acid transporter 1	cation transporter(PC00068)
GIGYF2	GRB10-interacting GYF protein 2		NTF3	Neurotrophin-3	neurotrophic factor(PC00163)
ZNF365	Protein ZNF365		RP1L1	Retinitis pigmentosa 1-like 1 protein	
THRB	Thyroid hormone receptor beta	C4 zinc finger nuclear receptor(PC00169);nucleic acid binding(PC00171);receptor(PC00197)	GABRB1	Gamma-aminobutyric acid receptor subunit beta-1	GABA receptor(PC00023);acetylcholine receptor(PC00037)
LRP2	Low-density lipoprotein receptor-related protein 2		COBL	Protein cordon-bleu	
RBPJ	Recombining binding protein suppressor of hairless	nucleic acid binding(PC00171);transcription factor(PC00218)	KIF13B	Kinesin-like protein KIF13B;KIF13B	microtubule binding motor protein(PC00156)
BOC	Brother of CDO		BRINP2	BMP/retinoic acid-inducible neural-specific protein 2	
TLR2	Toll-like receptor 2		GDNF	Glial cell line-derived neurotrophic factor	neurotrophic factor(PC00163)
PCDH15	Protocadherin-15		WNT5B	Protein Wnt-5b	signaling molecule(PC00207)
ADGRL3	Adhesion G protein-coupled receptor L3	G-protein coupled receptor(PC00021);antibacterial response protein(PC00051);protease(PC00190)	NTRK2	BDNF/NT-3 growth factors receptor	
ALCAM	CD166 antigen	immunoglobulin superfamily cell adhesion molecule(PC00125);receptor(PC00197)	CHL1	Neural cell adhesion molecule L1-like protein	
NTN4	Netrin-4	extracellular matrix linker protein(PC00101);protease inhibitor(PC00191);receptor(PC00197)	YAP1	Transcriptional coactivator YAP1	kinase modulator(PC00140);transcription cofactor(PC00217)
CPN1	Copine-1		PHGDH	D-3-phosphoglycerate dehydrogenase	dehydrogenase(PC00092)

ZEB2	Zinc finger E-box-binding homeobox 2	KRAB box transcription factor(PC00029)	OGDH	2-oxoglutarate dehydrogenase, mitochondrial	
CLASP2	CLIP-associating protein 2	structural protein(PC00211)	ARNTL	Aryl hydrocarbon receptor nuclear translocator-like protein 1	basic helix-loop-helix transcription factor(PC00055)
CDON	Cell adhesion molecule-related/down-regulated by oncogenes		CNTN6	Contactin-6	transmembrane receptor regulatory/adaptor protein(PC00226)
ARSB	Arylsulfatase B		MAGI2	Membrane-associated guanylate kinase, WW and PDZ domain-containing protein 2	
LHX6	LIM/homeobox protein Lhx6	RNA binding protein(PC00031)	ARID1B	AT-rich interactive domain-containing protein 1B	
DISC1	Disrupted in schizophrenia 1 protein		CPNE5	Copine-5	
CNTN1	Contactin-1		PBX1	Pre-B-cell leukemia transcription factor 1	homeodomain transcription factor(PC00119)
CSMD3	CUB and sushi domain-containing protein 3		COL25A1	Collagen alpha-1(XXV) chain	
DGKG	Diacylglycerol kinase gamma	kinase(PC00137)	KIDINS220	Kinase D-interacting substrate of 220 kDa	
WNK1	Serine/threonine-protein kinase WNK1	non-receptor serine/threonine protein kinase(PC00167)	NLGN1	Neuroligin-1	
KALRN	Kalirin	signaling molecule(PC00207)	CUX1	Homeobox protein cut-like 1	homeodomain transcription factor(PC00119);nucleic acid binding(PC00171)
NPY	Pro-neuropeptide Y	neuropeptide(PC00162)	SPOCK1	Testican-1	protease inhibitor(PC00191)
IGSF9	Protein turtle homolog A	transmembrane receptor regulatory/adaptor protein(PC00226)	IL6	Interleukin-6	
NEGR1	Neuronal growth regulator 1		DCDC2	Doublecortin domain-containing protein 2	
MEF2C	Myocyte-specific enhancer factor 2C	MADS box transcription factor(PC00250)	DRD2	D(2) dopamine receptor	G-protein coupled receptor(PC00021)
EYA1	Eyes absent homolog 1		OPCML	Opioid-binding protein/cell adhesion molecule	
FGFR1	Fibroblast growth factor receptor 1		MYPN	Myopalladin	

SEMA4D	Semaphorin-4D	membrane-bound signaling molecule(PC00152)	DNM3	Dynamin-3	hydrolase(PC00121);microtubule family cytoskeletal protein(PC00157);small GTPase(PC00208)
CHD7	Chromodomain-helicase-DNA-binding protein 7		SEMA5A	Semaphorin-5A	
TENM2	Teneurin-2		TNFRSF1B	Tumor necrosis factor receptor superfamily member 1B	
NRP1	Neuropilin-1		OLFM3	Noelin-3	receptor(PC00197);structural protein(PC00211)
PPARG	Peroxisome proliferator-activated receptor gamma	C4 zinc finger nuclear receptor(PC00169);nucleic acid binding(PC00171);receptor(PC00197)	PLXNA4	Plexin-A4	
NPHP4	Nephrocystin-4		NYAP2	Neuronal tyrosine-phosphorylated phosphoinositide-3-kinase adapter 2	
STK3	Serine/threonine-protein kinase 24		CDK1	Cyclin-dependent kinase 1	non-receptor serine/threonine protein kinase(PC00167);non-receptor tyrosine protein kinase(PC00168)
CNTN4	Contactin-4	transmembrane receptor regulatory/adaptor protein(PC00226)	WNT11	Protein Wnt-11	signaling molecule(PC00207)
PICALM	Phosphatidylinositol-binding clathrin assembly protein	vesicle coat protein(PC00235)	MATN2	Matrilin-2	receptor(PC00197)
LRRK2	Leucine-rich repeat serine/threonine-protein kinase 2		CDHR1	Cadherin-related family member 1	
NDNF	Protein NDNF		XRCC5	X-ray repair cross-complementing protein 5	DNA helicase(PC00011)
ASTN1	Astrotactin-1		PAX7	Paired box protein Pax-7	
GRIN3A	Glutamate receptor ionotropic, NMDA 3A		PTPRO	Receptor-type tyrosine-protein phosphatase O	
NAV1	Neuron navigator 1		C8orf37	Protein C8orf37	
CNTNAP2	Contactin-associated protein-like 2		SYT1	Synaptotagmin-1	membrane trafficking regulatory protein(PC00151)
APPL2	DCC-interacting protein 13-beta		TRIO	Triple functional domain protein	signaling molecule(PC00207)

HIPK2	Homeodomain-interacting protein kinase 2	non-receptor serine/threonine protein kinase(PC00167);non-receptor tyrosine protein kinase(PC00168)	SPINK5	Serine protease inhibitor Kazal-type 5	protease inhibitor(PC00191)
NRCAM	Neuronal cell adhesion molecule		VEGFC	Vascular endothelial growth factor C	growth factor(PC00112)
NOTCH1	Neurogenic locus notch homolog protein 1		PALLD	Palladin	
PRKG1	cGMP-dependent protein kinase 1		HDAC2	Histone deacetylase 2	deacetylase(PC00087);nucleic acid binding(PC00171);reductase (PC00198)
DNER	Delta and Notch-like epidermal growth factor-related receptor		RAC1	Ras-related C3 botulinum toxin substrate 1	small GTPase(PC00208)
ROBO2	Roundabout homolog 2		TENM3	Teneurin-3	
HECW1	E3 ubiquitin-protein ligase HECW1	ubiquitin-protein ligase(PC00234)	YWHAZ	14-3-3 protein zeta/delta	chaperone(PC00072)
BARHL1	BarH-like 1 homeobox protein	DNA binding protein(PC00009);homeodomain transcription factor(PC00119)	SCRIB	Protein scribble homolog	
SLIT3	Slit homolog 3 protein		FSTL4	Follistatin-related protein 4	protease inhibitor(PC00191)
MDGA1	MAM domain-containing glycosylphosphatidylinositol anchor protein 1		FSHR	Follicle-stimulating hormone receptor	extracellular matrix protein(PC00102);receptor(PC00197)
NCAM1	Neural cell adhesion molecule 1		ECT2	Protein ECT2	
SRRM4	Serine/arginine repetitive matrix protein 4		TNIK	TRAF2 and NCK-interacting protein kinase	
GRID2	Glutamate receptor ionotropic, delta-2		GRIP1	Glutamate receptor-interacting protein 1	
LMO4	LIM domain transcription factor LMO4		PRKCQ	Protein kinase C theta type	annexin(PC00050);calmodulin(PC00061);non-receptor serine/threonine protein kinase(PC00167);transfer/carrier protein(PC00219)
RIMS1	Regulating synaptic membrane exocytosis protein 1	G-protein modulator(PC00022)	SLIT1	Slit homolog 1 protein	
LZTS1	Leucine zipper putative tumor suppressor 1		RTN4IP1	Reticulon-4-interacting protein 1, mitochondrial	

NFIB	Nuclear factor 1 B-type	nucleic acid binding(PC00171);transcription factor(PC00218)	UNC5C	Netrin receptor UNC5C	receptor(PC00197)
TERT	Telomerase reverse transcriptase	reverse transcriptase(PC00200)	ALK	ALK tyrosine kinase receptor	
IGSF10	Immunoglobulin superfamily member 10		LMX1A	LIM homeobox transcription factor 1-alpha	RNA binding protein(PC00031)
ST7	Low-density lipoprotein receptor-related protein 12	metalloprotease(PC00153)	BCL11A	B-cell lymphoma/leukemia 11A	C2H2 zinc finger transcription factor(PC00248)
NFASC	Neurofascin		HECW2	E3 ubiquitin-protein ligase HECW2	ubiquitin-protein ligase(PC00234)
CDH23	Cadherin-23		GFRA1	GDNF family receptor alpha-1	
SOX5	Transcription factor SOX-5		SHANK2	SH3 and multiple ankyrin repeat domains protein 2	
CRMP1	Dihydropyrimidinase-related protein 1	metalloprotease(PC00153)	UBE4B	Ubiquitin conjugation factor E4 B	enzyme modulator(PC00095)
TRIM67	Tripartite motif-containing protein 67		HTT	Sodium-dependent serotonin transporter	cation transporter(PC00068)
FARP2	FERM, ARHGEF and pleckstrin domain-containing protein 2		TNR	Tenascin-R	signaling molecule(PC00207)
EPHB2	Ephrin type-B receptor 2		MAP1B	Microtubule-associate d protein 1B	non-motor microtubule binding protein(PC00166)
CAMK1 D	Calcium/calmodulin-depend ent protein kinase type 1D	non-receptor serine/threonine protein kinase(PC00167)	RAP1GAP	Rap1 GTPase-activating protein 1	G-protein modulator(PC00022)
GLI3	Transcriptional activator GLI3		VWC2	Brorin	
UBA6	Ubiquitin-like modifier-activating enzyme 6	ligase(PC00142);transfer/carrier protein(PC00219)	APBB2	Amyloid-beta A4 precursor protein-binding family B member 2	
DGUOK	Deoxyguanosine kinase, mitochondrial	nucleotide kinase(PC00172)	NRXN1	Neurexin-1	
SRGAP2	SLIT-ROBO Rho GTPase-activating protein 2	G-protein modulator(PC00022)	SEMA6A	Semaphorin-6A	membrane-bound signaling molecule(PC00152)
COL3A1	Collagen alpha-1(III) chain		SERPINE 2	Glia-derived nexin	serine protease inhibitor(PC00204)
MYT1L	Myelin transcription factor 1-like protein	zinc finger transcription factor(PC00244)	TRAPPC9	Trafficking protein particle complex subunit 9	

DSCAML1	Down syndrome cell adhesion molecule-like protein 1		SEMA3C	Semaphorin-3C	membrane-bound signaling molecule(PC00152)
SEMA3E	Semaphorin-3E	membrane-bound signaling molecule(PC00152)	LRP6	Low-density lipoprotein receptor-related protein 6	
LMX1B	LIM homeobox transcription factor 1-beta	RNA binding protein(PC00031)	ADGRB3	Adhesion G protein-coupled receptor B3	G-protein coupled receptor(PC00021);antibacterial response protein(PC00051);protease(PC00190)
BMP6	Bone morphogenetic protein 6	growth factor(PC00112)	TMEM108	Transmembrane protein 108	
TENM4	Teneurin-4;TENM4;ortholog		KIRREL3	Kin of IRRE-like protein 3	
NRG3	Pro-neuregulin-3, membrane-bound isoform	growth factor(PC00112)	NRP2	Neuropilin-2	
FLRT1	Leucine-rich repeat transmembrane protein FLRT1		USH2A	Usherin	extracellular matrix linker protein(PC00101);protease inhibitor(PC00191);receptor(PC00197)
NRG1	Pro-neuregulin-1, membrane-bound isoform	growth factor(PC00112)	VAX2	Ventral anterior homeobox 2	DNA binding protein(PC00009);homeodomain transcription factor(PC00119)
SCARB2	Lysosome membrane protein 2	receptor(PC00197)	BMPR1B	Bone morphogenetic protein receptor type-1B	TGF-beta receptor(PC00035);serine/threonine protein kinase receptor(PC00205)
CDK6	Cyclin-dependent kinase 6	non-receptor serine/threonine protein kinase(PC00167);non-receptor tyrosine protein kinase(PC00168)	PTK2B	Protein-tyrosine kinase 2-beta	
FGFR2	Fibroblast growth factor receptor 2		PTN	Pleiotrophin	cytokine(PC00083)
EXT1	Exostosin-1	glycosyltransferase(PC00111)	DAB2IP	Disabled homolog 2-interacting protein	G-protein modulator(PC00022)
DAB1	Disabled homolog 1	signaling molecule(PC00207)	ATP2B2	Plasma membrane calcium-transporting ATPase 2	cation transporter(PC00068);hydrolase(PC00121);ion channel(PC00133)
DOCK10	Dedicator of cytokinesis protein 10	guanyl-nucleotide exchange factor(PC00113)	CTNND2	Catenin delta-2	intermediate filament binding protein(PC00130)
OPRM1	Mu-type opioid receptor	G-protein coupled receptor(PC00021)	ABCC8	ATP-binding cassette sub-family C member 8	ATP-binding cassette (ABC) transporter(PC00003)

Dec1	Class E basic helix-loop-helix protein 40	basic helix-loop-helix transcription factor(PC00055)	ASAP1	Arf-GAP with SH3 domain, ANK repeat and PH domain-containing protein 1	
ERBB4	Receptor tyrosine-protein kinase erbB-4		SDK1	Protein sidekick-1	
ANK3	Ankyrin-3		WNT2B	Protein Wnt-2b	signaling molecule(PC00207)

Principal Component Analysis

Figure S1 shows an ensemble of plots detailing the population structure along with the distributions of responders and non-responders in the training and validation sets in each fold for the aggregate analysis. The y-axis in each subplot represents the first principal component (PC) with each column representing PCs two, three and four respectively. Each plot in the first row has points colored by data collection site where circular points represent non-responders and crosses represent responders. In this first row we see a clear representation of the population structure that exists between sites. In each of the next five rows of plots each point is again shaped according to lithium responsiveness, but is colored according to whether it was in the training or validation set for the given fold. If population structure interferes with assessment of performance in the aggregate analysis, then one should appreciate a systematic relationship between *all three of* (A) population structure, (B) lithium response, and (C) inclusion in all validation sets.

From Figure S1 we see that, despite the existence of population structure and variance in class proportions between sites, there is an effectively random distribution of training and validation examples across folds, which does not allow a classifier to consistently exploit population structure during cross validation.

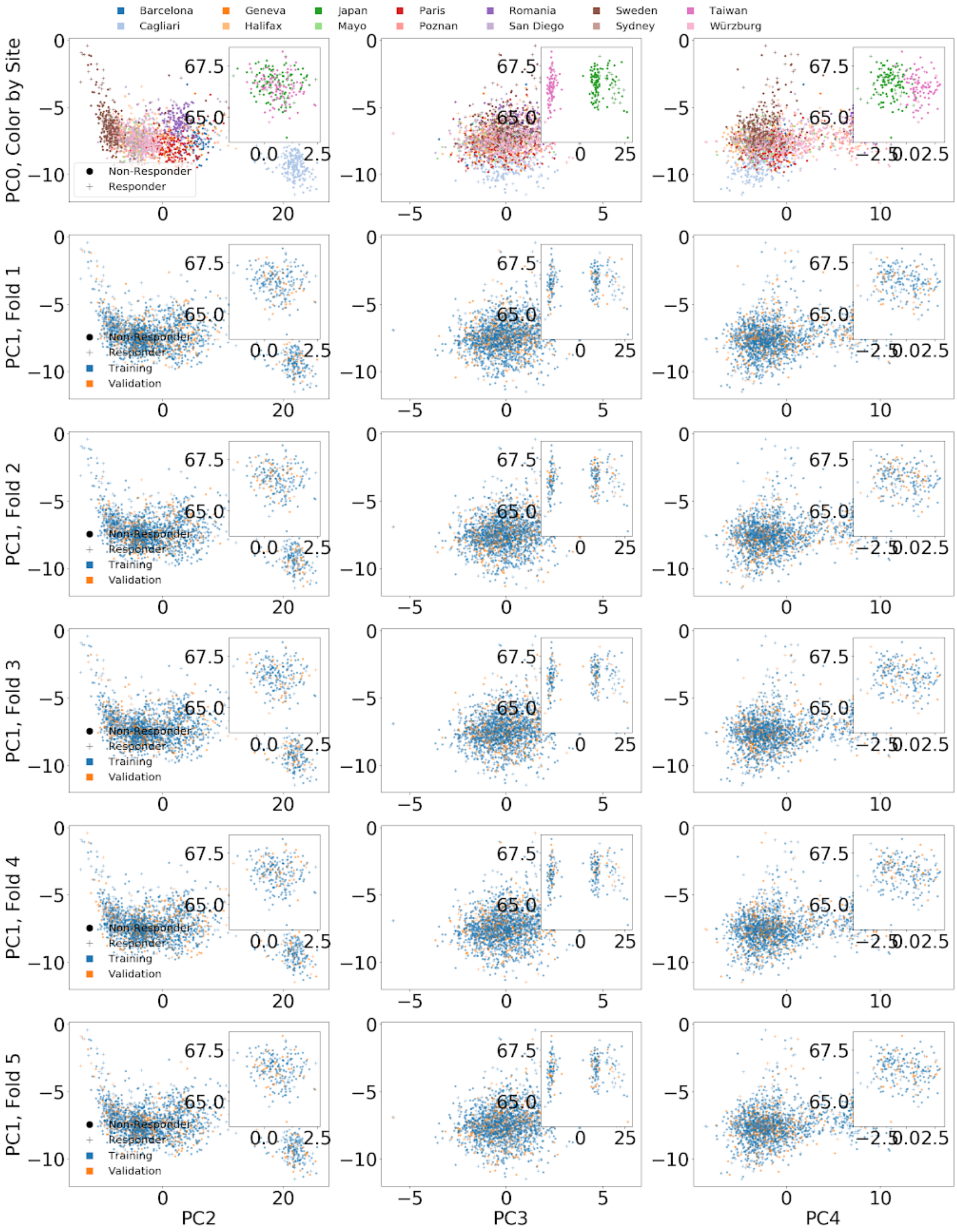

Figure S3: This figure shows the first principal component (PC) of the genotype matrix plotted against the 2nd, 3rd and 4th PCs with various coloring schemes. In each subplot the shape of the points represents whether each sample is a lithium responder (cross) or non-responder (circle). In the first row, samples are colored according to which site they originated from. In each of the next five rows the color represents

whether or not the sample was in the training or validation set for each fold.

References

1. Grof P, Duffy A, Cavazzoni P, Grof E, Garnham J, MacDougall M, et al. Is response to prophylactic lithium a familial trait? J Clin Psychiatry. 2002;63(10):942–947.

2. Manchia, M., Adli, M., Akula, N., Ardau, R., Aubry, J. M., Backlund, L., et al. Assessment of response to lithium maintenance treatment in bipolar disorder: a Consortium on Lithium Genetics (ConLiGen) report. *PloS one*, 2013;*8*(6).
